# Constituents of *Gastrodia elata* and Their Neuroprotective Effects in HT22 Hippocampal Neuronal, R28 Retinal Cells, and BV2 Microglial Cells

**DOI:** 10.3390/plants9081051

**Published:** 2020-08-18

**Authors:** Hye Mi Kim, Jaeyoung Kwon, Kyerim Lee, Jae Wook Lee, Dae Sik Jang, Hak Cheol Kwon

**Affiliations:** 1 College of Pharmacy, Kyung Hee University, Seoul 02447, Korea; hyemi586@gmail.com; 2 KIST Gangneung Institute of Natural Products, Korea Institute of Science and Technology (KIST), Gangneung 25451, Korea; kjy1207@kist.re.kr (J.K.); klim8@kist.re.kr (K.L.); jwlee5@kist.re.kr (J.W.L.); 3 KHU-KIST Department of Converging Science and Technology, Kyung Hee University, Seoul 02447, Korea

**Keywords:** *Gastrodia elata*, phenolic compound, tripeptide, neuroprotection, neuronal cell, microglial cell

## Abstract

*Gastrodia elata* is widely used in traditional medicine and contains various types of metabolites with pharmacological activity. In the course of searching for neuroprotective molecules associated with the potential of *G. elata* in the treatment of neurodegenerative disorders, two new phenolic compounds (**1** and **2**) and a new tripeptide (**3**), together with 16 known compounds (**4**–**19**), were isolated from the rhizomes of *G. elata*. The structures of the compounds were determined by the interpretation of spectroscopic data, including nuclear magnetic resonance and mass spectrometry data. All obtained compounds were assessed for their ability to protect neuronal cells against neurotoxicity and neuroinflammation. Of these, **4** and **5** were found to possess moderate activities in HT22 hippocampal neuronal cells, whereas **2**, **6**, and **7** showed weak activities in R28 retinal cells. Additionally, compound **9** showed moderate inhibitory activity on lipopolysaccharide-induced nitric oxide production in BV2 microglial cells.

## 1. Introduction

Oxidative stress is implicated in several neurological and ophthalmic diseases, including Alzheimer’s disease, Parkinson’s disease, ischemic stroke, and glaucoma [1,2]. Glutamate or hydrogen peroxide (H_2_O_2_) toxicity is a common model used to study oxidative stress-induced neuronal or optic neuronal cell death, which is associated with acute and chronic cellular insults [1]. In neuronal cells, a high concentration of extracellular glutamate can inhibit the production of glutathione (GSH) by disturbing the uptake of cystine into cells, and leading to the accumulation of reactive oxygen species (ROS). An increase in intracellular ROS induces neuronal cell death by multiple pathways, including apoptosis, necrosis, ferroptosis, or other forms of cell death [3].

Retina cells are a type of neuron that have originated from a similar lineage of neuronal cells. Retina tissue is high oxygen-consuming and converts visual information into electronic signals that can transfer from the eye to the brain. An increase in oxygen consumption can induce oxidative stress that, in turn, can induce damage to the retina and represents a risk factor for several retinal disorders, including glaucoma [4,5]. Therefore, protecting neuronal and retina cells from oxidative stress is a major therapeutic strategy in preventing the pathogenesis of oxidative stress-related neurological and ophthalmic diseases [6].

Besides, the modulation of nitric oxide (NO) secretion in microglia cells can be beneficial to neurodegenerative diseases. The microglia cell is a main glial cell type that plays pivotal roles in the innate immune response and phagocytosis that can remove cell debris and damaged neurons. In disease conditions, microglia activated by lipopolysaccharide (LPS) significantly accelerates neuroinflammation which releases cyclooxygenase-2, NO synthase as well as interleukin-1b and -6, tumor necrosis factor-α, and NO. In particular, a high level of NO can induce oxidative stress and neuron destruction contributing to a neurodegenerative disorder [7].

*Gastrodia elata* Blume (family Orchidaceae), commonly known as tianma, has been used for the treatment of neurological disorders such as headache, dizziness, limb numbness, infantile convulsion, vertigo, epilepsy, and tonic spasm [8]. Previous phytochemical investigations of *G. elata* have revealed the presence of various phenolic compounds, sterols, and organic acids, which exhibit a range of biological activities, including neuroprotective, antioxidant, anti-inflammatory, and anticonvulsant effects [9,10,11,12]. In particular, several phenolic compounds, including gastrodin, gastrodigenin, and vanillin, have been shown to protect neuronal cells and restore the function of the brain by suppressing oxidative stress and inflammatory responses [13].

These results have encouraged further studies in an attempt to discover natural neuroprotective molecules from the rhizomes of *G. elata*. Nineteen compounds, including two new phenolic compounds (**1** and **2**) and a new tripeptide (**3**), were isolated and structurally characterized from *n*-hexane-, ethyl acetate (EtOAc)-, and water (H_2_O)-soluble fractions of the rhizomes of *G. elata*. The neuroprotective and optic nerve-protective effects of all isolated compounds were examined against glutamate- or H_2_O_2_-induced toxicity in HT22 hippocampal neuronal and R28 retinal cells, respectively. In addition, all obtained compounds were also assessed for their anti-neuroinflammatory effects in BV2 microglial cells through inhibiting LPS-induced NO production.

Herein, this paper describes the structural identification of three new compounds (**1**–**3**) and biological assessment of the compounds associated with the potential of *G. elata* in the treatment of neurodegenerative disorders.

## 2. Results and Discussion

### 2.1. Isolation and Identification of Compounds from the Rhizomes of G. elata

**Figure 1:**
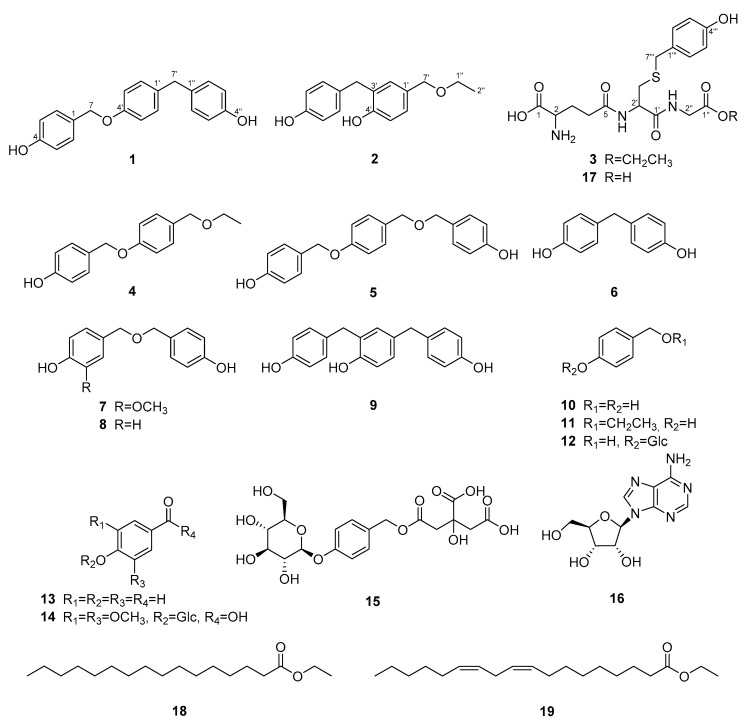
Chemical structures of compounds **1**–**19** isolated from the rhizomes of *G. elata*.

Compound **1** was isolated as a yellowish amorphous powder, and its molecular formula, C_20_H_18_O_3_, was determined by high-resolution mass spectrometry (HRMS) data (Figure S8). The ultraviolet (UV) spectrum (Figure S6) exhibited maximum absorption at 227 and 281 nm, and the infrared (IR) spectrum (Figure S7) displayed absorption bands at 3334, 1228, 1105, and 1014 cm^−1^, indicating that **1** is a phenolic compound with hydroxy and ether groups. The ^1^H nuclear magnetic resonance (NMR) data (Table 1 and Figure S1) showed three pairs of AA′BB′ type signals at *δ*_H_ 7.28 (2H, d, *J* = 8.5 Hz, H-2, H-6), 6.84 (2H, d, *J* = 8.5 Hz, H-3, H-5), 7.10 (2H, d, *J* = 8.5 Hz, H-2′, H-6′), 6.89 (2H, d, *J* = 8.5 Hz, H-3′, H-5′), 7.02 (2H, d, *J* = 8.0 Hz, H-2″, H-6″), and 6.74 (2H, d, *J* = 8.0 Hz, H-3″, H-5″), as well as two methylene signals at *δ*_H_ 4.94 (2H, s, H-7) and 3.80 (2H, s, H-7′). The ^13^C NMR data (Table 1 and Figure S2) displayed 20 carbon signals, suggesting the presence of three sets of 1,4-disubstituted phenol groups. These assignments were supported by a detailed analysis of the two-dimensional (2D) NMR spectra (Figures S3 and S5). The heteronuclear multiple bond correlation (HMBC) cross-peaks from H-7 to C-2 (*δ*_C_ 130.3) and C-4′ (*δ*_C_ 158.2), as well as the cross-peaks from H-7′ to C-2′ (*δ*_C_ 130.4) and C-2″ (*δ*_C_ 130.5), indicated the location of each phenol group. Consequently, the structure of compound **1** was determined to be 4-[[4-(4-hydroxybenzyl)phenoxy]methyl]phenol (Figure 1).

Compound **2** was isolated as a yellowish oil. The molecular formula, C_16_H_18_O_3_, was determined by HRMS data. The ^1^H and ^13^C NMR data of **2** (Table 1) were superimposable to those of the already reported compound, 4-hydroxy-3-(4-hydroxybenzyl)benzyl methyl ether [14], except for the replacement of a methoxy group at C-7′ by an ethoxy group. The ^1^H NMR signals of the ethoxy group appeared at *δ*_H_ 3.57 (2H, q, *J* = 7.0 Hz, H-1″) and 1.24 (3H, t, *J* = 7.0 Hz, H-2″). Its connectivity was demonstrated by the HMBC cross-peak from H-1″ to C-7′ (*δ*_C_ 72.8). Furthermore, an additional interpretation of the 2D NMR suggested that compound **2** is 4-(ethoxymethyl)-2-(4-hydroxybenzyl) phenol.

Compound **3** was isolated as a pale yellowish amorphous powder. The UV and IR data of **3** were almost the same as those of **17**, indicating that the compounds possess a similar skeleton, *S*-(4-hydroxybenzyl)glutathione. The ^1^H NMR data of **3** were superimposable to that of **17**, with the only difference being the presence of an additional ethoxy group at *δ*_H_ 4.16 (2H, q, *J* = 7.0 Hz) and 1.25 (3H, t, *J* = 7.0 Hz), as further supported by HRMS data. Furthermore, the basic skeleton was reconfirmed by ^1^H–^1^H correlation spectroscopy (COSY) and HMBC data. In particular, the connectivity between glutathione and 4-hydroxybenzyl groups was established by the HMBC correlation from H-3′ to C-7‴ (*δ*_C_ 36.5), suggesting that the 4-hydroxybenzyl group was connected to the sulfur atom of the cysteinyl unit, and the location of the ethoxy group was determined by the HMBC correlation from the methylene proton signal at *δ*_H_ 4.16 to C-1″ (*δ*_C_ 171.1). Therefore, the structure of compound **3** was elucidated as ethyl *S*-(4-hydroxybenzyl) glutathione.

The previously reported compounds were identified to be 4-[[4-(ethoxymethyl)phenoxy] methyl]phenol (**4**) [15], gastrol A (**5**) [16], bis(4-hydroxyphenyl)methane (bisphenol F) (**6**) [10], 4-hydroxybenzyl vanillyl ether (**7**) [17], bis(4-hydroxybenzyl)ether (**8**) [18], 2,4-bis(4-hydroxybenzyl)phenol (**9**) [10], gastrodigenin (**10**) [18], 4-hydroxybenzyl ethyl ether (**11**) [19], gastrodin (**12**) [18], 4-hydroxybenzaldehyde (**13**) [18], 3,5-dimethoxybenzoic acid-4-*O*-*β*-d-glucopyranoside (**14**) [20], parishin E (**15**) [19], adenosine (**16**) [21], *S*-(4-hydroxybenzyl)glutathione (**17**) [22], palmitic acid ethyl ester (**18**) [23], and linoleic acid ethyl ester (**19**) [23] by comparing the UV, IR, and NMR spectroscopic data with published values.

Although ethylated metabolites have been reported in *G. elata*, as in the example of compounds **4** and **11**, compound **2** may represent artifacts produced during the ethanol (EtOH) extraction process [8,22]. However, the purpose of this study is to evaluate the value of the EtOH extract of *G. elata* as a neuroprotective agent by identifying bioactive compounds extracted from EtOH. Therefore, if effective and reproducible, ethylated constituents of the *G. elata* EtOH extract may also be suitable for the purposes of this study.

### 2.2. Neuroprotective and NO Inhibitory Effects of Compounds from the Rhizomes of G. elata

**Figure 2:**
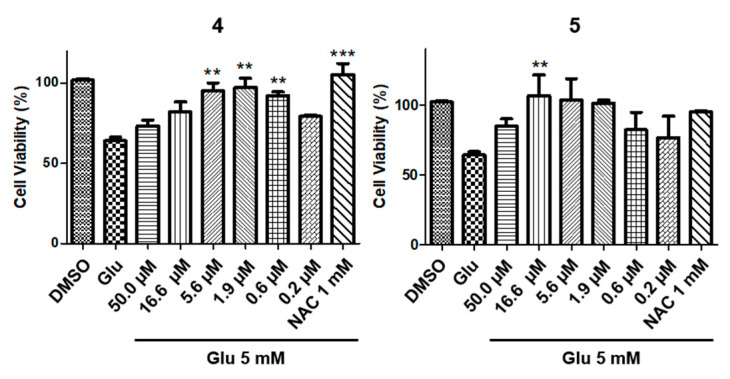
Protective effect of **4** and **5** against glutamate-induced toxicity in HT22 cells (** *P* < 0.05, and *** *P* < 0.0002).

To discover potent neuroprotective compounds, all obtained compounds **1**–**19**, isolated from *G. elata*, were assessed for their activities against glutamate-induced toxicity in HT22 cells (Table S1). The HT22-immortalized mouse hippocampal neuronal cell line has commonly been used to evaluate the ability of compounds to protect neuronal cells [24]. *N*-acetyl-l-cysteine (NAC) was used as a positive control. Among the 19 compounds, compounds **4** and **5** showed more than 90% cell viability and were further analyzed for dose-dependency using a six-point concentration series. Even though cell viability was decreased to 60% with 5 mM of glutamate, treatment with a concentration ranging from 5.6 to 0.6 μM of **4**, or a concentration ranging from 16.7 to 0.6 μM of **5**, was associated with a protective effect of ≥90%, measured as cell viability, against glutamate-induced toxicity (Figure 2). Both an effective concentration and cell viability are regarded as important factors in a cell protection assay.

**Figure 3:**
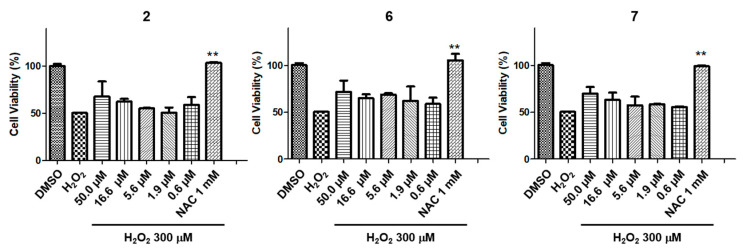
Protective effects of **2, 6**, and **7** against H_2_O_2_-induced toxicity in R28 cells (** *P* < 0.005).

R28 retinal cells were used to investigate protective compounds for optical neurons [25]. Among the isolated compounds, three compounds (**2**, **6**, and **7**) were selected through a screening system (Table S2) to be further studied using a six-point concentration series. NAC was used as a positive control. After the addition of 300 μM of H_2_O_2_, cell viability decreased to 50% due to oxidative stress-induced cell death but was restored to 60%-70% after treatment with with 50 to 16.6 μM of either **2**, **6**, or **7** (Figure 3).

**Figure 4:**
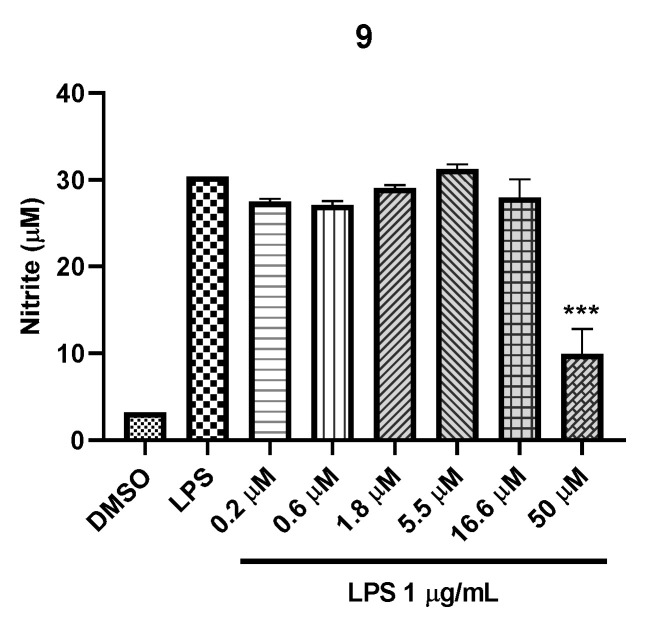
Inhibitory effect of **9** against LPS-induced nitric oxide production in BV2 cells (*** *P* < 0.001).

Besides, the inhibitory effects of compounds isolated from the EtOH extract of *G. elata* on NO production were evaluated in LPS-treated murine BV2 microglial cells at a concentration ranging from 0.02 to 50.0 μM (Table S3). The cytotoxicity of these compounds was measured using the MTT assay. Inhibition of NO production in LPS-induced microglial cells is considered an effective approach to alleviating the inflammatory response in neurons [26]. Only compound **9** showed moderate NO inhibitory activities with IC_50_ 38.98 μM, while **9** showed no cytotoxicity at all concentrations treated in the cell lines (50 to 0.2 μM) (Figure 4). 

Although the compound **6** possesses potential bioactivity for neuroprotection, its neurotoxicity toward a zebrafish embryos was reported [27], so this potential toxicity should be considered for the use of *G. elata* extract or bisphenol F (**6**). However, the toxicity of bisphenol F (**6**) might be much weaker than that of bisphenol A. Bisphenol A could cause developmental abnormalities in the neuronal system through possibly inducing degradation of hypoxia-inducible factor 1α (HIF-1α). A recent report has documented that the HIF-1α-related toxicity of bisphenol A disappeared by the removal of two central methyl groups in bisphenol A to obtain bisphenol F [28].

Although the neuroprotective effect of gastrodin (**12**) on glutamate-treated PC12 cells and H_2_O_2_-treated SH-SY5Y cells was reported, it was not effective in our toxicity-inducing model using glutamate in HT22 cells or H_2_O_2_ in R28 cells [29,30]. Gastrodin, a major phenolic component of *G. elata*, can prevent hypoxia-induced neurotoxicity in cultured rat cortical neurons [31] and has shown antioxidative and neuroprotective effects in a transient focal brain ischemia model in the rodent [32]. In our bioassay model for LPS-induced nitric oxide inhibition, gastrodin showed no activity at 50 μM. However, it is reported that the treatment of gastrodin (40 μM and 60 μM) can inhibit LPS-induced enhancement of expression of the iNOS in microglial cells [33]. Although 4-hydroxybenzaldehyde (**13**) was not effective in our neuroprotection assay either, it possesses an antioxidative effect on brain lipid peroxidation in pentylenetetrazole-induced convulsions in rats [34] and can inhibit glutamate-induced apoptosis in IMR-32 neuroblastoma cells [35].

The neuroprotective effects of compounds **2**, **4**, **5**, **6** and **7** on H_2_O_2_ or glutamate-induced toxicity in hippocampal and retinal cells are first reported in this study. The inhibition of **9** on LPS-induced nitric oxide production was not reported in the previous literature either. This present study was limited to the discovery of additional neuroprotective compounds derived from *G. elata*. In order to classify the various neuroprotective constituents of *G. elata* according to their biological activity range and investigate the potentials for drug development, additional structure-activity relationship (SAR), mechanism of action (MOA) and in vivo efficacy studies of bioactive compounds from this study should be conducted.

## 3. Materials and Methods 

### 3.1. General Procedures

UV spectra were obtained on a Spectramax M5 (Molecular Devices). IR spectra were obtained using a FT/IR-4200 spectrometer (Jasco). HRMS data were acquired using a Waters xevo G2 quadrupole time-of-flight (QTOF) mass spectrometer equipped with electrospray ionization. NMR experiments were conducted on a Varian 500 MHz and a Jeol 500 MHz using tetramethylsilane or solvent residues as reference signals, and the chemical shifts were expressed as δ values. Thin-layer chromatography (TLC) analyses were performed on silica gel 60 F_254_ plates and RP-18 F_254S_ plates (0.25 mm, Merck), and compounds were visualized by UV light (254 and 365 nm) and 20% (*v/v*) H_2_SO_4_ reagent (Sigma-Aldrich). Silica gel (70–230 or 230–400 mesh, Merck), Sephadex LH-20 (Amersham Pharmacia Biotech), and Diaion HP-20 (Mitsubishi Chemical Co.) were used for column chromatography. Prepacked cartridges, Redi Sep-C_18_ (13 g, 26 g, 43 g, 130 g, Teledyne Isco), were used for flash chromatography. Semi-preparative high-performance liquid chromatography (HPLC) was performed using a Gilson 321 pump and a Gilson 155 UV/VIS detector, with a YMC Pack ODS-A column (5 μm, 250 × 20 mm i.d.). All solvents used for the chromatographic separations were distilled before use.

### 3.2. Plant Material

The rhizomes of *G. elata* Blume (Orchidaceae) were obtained from a domestic Korean market (Muju Firefly Market, Muju-gun, Jeollabuk-do), in April 2015. The origin of the herbal material was identified by Prof. Dae Sik Jang and a voucher specimen (GAEL1-2015) has been deposited in the Lab. of Natural Product Medicine, College of Pharmacy, Kyung Hee University, Republic of Korea.

### 3.3. Extraction and Isolation

The rhizomes of fresh *G. elata* (17.4 kg) were extracted with EtOH (35 L) twice at room temperature. The EtOH extract was partitioned with *n*-hexane (18 L × 2) and EtOAc (18 L × 2) to yield *n*-hexane- (5.8 g), EtOAc- (36.3 g), and H_2_O-soluble extracts (500.0 g), respectively.

The *n*-hexane-soluble extract (5.8 g) was subjected to silica gel column chromatography (CC) (70–230 mesh, ø 4.5 × 45.0 cm) using *n*-hexane–EtOAc–methanol (MeOH) mixtures (19:1:0 to 0:0:1, *v/v*) as the mobile phase to produce 18 fractions (H1 to H14). Fraction H2 (1.1 g) was further fractionated using silica gel CC (230-400 mesh, ø 3.5 × 27.0 cm) eluted with *n*-hexane–EtOAc (49:1 to 0:1, *v/v*) to produce compounds **18** (135.5 mg, yield 0.0249% by weight of the ethanol extract) and **19** (15.3 mg, yield 0.0028%).

The EtOAc-soluble extract (36.3 g) was chromatographed over silica gel CC (70–230 mesh, ø 4.7 × 45.0 cm) with dichloromethane (CH_2_Cl_2_)–MeOH mixtures (19:1 to 0:1, *v/v*) to produce 15 fractions (E1 to E15). Fraction E2 (7.1 g) was subjected to silica gel CC (230–400 mesh, ø 4.9 × 34.9 cm) with *n*-hexane–EtOAc mixtures (7:3 to 3:2, *v/v*) to produce 14 subfractions (E2-1 to E2-14). Subfraction E2-3 (4.7 g) was further fractionated to 13 subfractions (E2-3-1 to E-2-3-13) using silica gel (230–400 mesh, ø 4.3 × 37.9 cm) with *n*-hexane–EtOAc mixtures (7:3 to 0:1, *v/v*). Subfractions E2-3-4 (2.1 g) and E2-3-5 (75.1 mg) were separated by a flash chromatography system using Redi Sep-C_18_ cartridges (130 g, MeOH–H_2_O, 3:7 to 7:3, *v/v*; 26 g, MeOH–H_2_O, 3:7 to 4:1, *v/v*), respectively, to obtain compound **4** (121.4 mg, yield 0.0223%). Subfraction E2-6 (570.1 mg) was separated by a flash chromatography system using Redi Sep-C_18_ (43 g, MeOH–H_2_O, 3:7 to 7:3, *v/v*), followed by preparative HPLC with a YMC Pack ODS-A column to obtain compound **1** (10.7 mg, yield 0.0020%). Fraction E3 (2.4 g) was fractionated using silica gel CC (230–400 mesh, ø 3.7 × 26.3 cm) with *n*-hexane–EtOAc (7:3 to 3:2, *v/v*) to produce 13 subfractions (E3-1 to E3-13). Subfractions E3-2 (729.3 mg) and E3-5 (725.4 mg) were separated using a flash chromatography system and Redi Sep-C_18_ cartridges (43 g, MeOH–H2O, 1:3 to 9:11, *v/v*; 130 g, MeOH–H_2_O, 3:7 to 1:1, *v/v*), respectively, to obtain compounds **2** (178.7 mg, yield 0.0328%), **6** (248.9 mg, 0.0457%), **11** (247.5 mg, 0.0454%), and **13** (14.1 mg, 0.00259%). Subfraction E3-7 (216.9 mg, yield 0.0398%) was separated on a flash chromatography system with Redi Sep-C_18_ (26 g, MeOH–H2O, 1:3 to 1:1, *v/v*), followed by preparative HPLC with a YMC Pack ODS-A column, to yield compounds **5** (2.7 mg, yield 0.0005%), **7** (2.6 mg, 0.0005%), **8** (6.4 mg, 0.0012%), and **10** (13.7 mg, 0.0025%). Fraction E5 (1.8 g) was separated using silica gel CC (230–400 mesh, ø 3.6 × 27.5 cm, *n*-hexane–EtOAc, 3:2 to 0:1, *v/v*) to produce 13 subfractions (E5-1 to E5-13). Subfractions E5-4 (118.6 mg) and E5-5 (187.5 mg) were separated through the flash chromatography system using a Redi Sep-C_18_ cartridge (26 g, MeOH–H_2_O, 1:1 to 3:1, v/v) to obtain compounds **9** (68.9 mg, yield 0.0126%) and **10** (35.8 mg, 0.0066%), respectively.

The H_2_O-soluble extract (500.0 g) was chromatographed over Diaion HP-20 CC (ø 8.0 × 57.0 cm) and eluted with MeOH–H_2_O mixtures (0:1 to 1:0, *v/v*) to produce 18 fractions (W1 to W18). Fraction W5 (8.1 g) was further fractionated using Sephadex LH-20 (ø 5.0 × 53.5 cm) with MeOH–H_2_O mixtures (3:7 to 1:0, *v/v*), followed by preparative HPLC with a YMC Pack ODS-A column, resulting in the isolation of compounds **12** (8.9 mg, yield 0.0016%) and **15** (14.5 mg, 0.0027%). Fraction W8 (5.2 g) was further subjected to Sephadex LH-20 (ø 4.6 × 51.0 cm) with MeOH–H_2_O mixtures (2:3 to 1:0, *v/v*) to produce eight subfractions (W8-1 to W8-8). Subfraction W8-5 (1.46 g) was fractionated using silica gel CC (230–400 mesh, ø 3.6 × 26.0 cm) with EtOAc–MeOH–H_2_O mixtures (40:9:1 to 0:1:0, *v/v*) to produce nine subfractions (W8-5-1 to W8-5-9). Compound **16** (143.2 mg, yield 0.0263%) was purified by recrystallization with MeOH from subfraction W8-5-4 (28.4 mg). Subfractions W8-5-5 (73.3 mg) and W8-5-6 (62.5 mg) were separated by a flash chromatography system with Redi Sep-C_18_ (13 g, MeOH–H_2_O, 0:1 to 7:3, *v/v*), followed by preparative HPLC with a YMC Pack ODS-A column, to obtain compounds **3** (3.6 mg, yield 0.0007%) and **14** (4.8 mg, 0.0009%). Subfraction W8-5-9 (790.0 mg) was purified using a flash chromatography system and Redi Sep-C_18_ (43 g, MeOH–H_2_O, 0:1 to 1:1, *v/v*), and recrystallized with MeOH, resulting in the isolation of compound **17** (233.3 mg, yield 0.0428%).

#### 3.3.1. 4-[[4-(4-Hydroxybenzyl)phenoxy]methyl]phenol (**1**)

Yellowish amorphous powder; UV (MeOH); λ_max_ (log *ε*) 227 (4.60), 281 (4.00) nm; IR (ATR) *ν*_max_ 3334, 1611, 1509, 1228, 1105, 1014, 820 cm^−1^; ^1^H and ^13^C NMR data (500 and 125 MHz, CD_3_COCD_3_), see Table 1; HRESIMS (negative mode) *m*/*z* 305.1174 [M − H]^−^ (calcd. for C_20_H_17_O_3_, 305.1178).

#### 3.3.2. 4-(Ethoxymethyl)-2-(4-hydroxybenzyl)phenol (**2**)

Yellowish oil; UV (MeOH); *λ*_max_ (log *ε*) 229 (4.53), 280 (3.90) nm; IR (ATR) *ν*_max_ 3324, 1612, 1511, 1236, 1069, 820 cm^−1^; ^1^H and ^13^C NMR data (500 and 125 MHz, CDCl_3_), see Table 1; HRESIMS (negative mode) *m*/*z* = 257.1177 [M − H]^−^ (calcd. for C_16_H_17_O_3_, 257.1178).

#### 3.3.3. Ethyl S-(4-hydroxybenzyl) Glutathione (**3**)

Pale yellowish amorphous powder; *λ*_max_ (log *ε*) 228 (5.21), 279 (4.43) nm; IR (ATR) *ν*_max_ 3273, 2981, 2938, 1746, 1647, 1514, 1451, 1415, 1375, 1339, 1216, 1032, 835 cm^−1^; ^1^H NMR (500 MHz, CD_3_OD) δ 1.25 (3H, t, *J* = 7.0 Hz, CH_2_CH_3_), 2.07 (1H, m, H_2_-3), 2.15 (1H, m, H_2_-3), 2.51 (2H, m, H_2_-4), 2.67 (1H, dd, *J* = 13.5, 9.0 Hz, H_2_-3′), 2.91 (1H, dd, *J* = 13.5, 5.5 Hz, H_2_-3′), 3.63 (1H, dd, *J* = 6.5, 6.5 Hz, H-2), 3.68 (2H, s, H_2_-7‴), 3.89 (1H, d, *J* = 17.5 Hz, H_2_-2″), 3.95 (1H, d, *J* = 17.5 Hz, H_2_-2″), 4.16 (2H, q, *J* = 7.0 Hz, CH_2_CH_3_), 4.55 (1H, dd, *J* = 9.0, 5.5 Hz, H-2′), 6.72 (2H, d, *J* = 8.5 Hz, H-3‴, H-5‴), 7.15 (2H, d, *J* = 8.5 Hz, H-2‴, H-6‴); ^13^C NMR (125 MHz, CD_3_OD) δ 14.5 (CH_2_CH_3_), 27.8 (C-3), 32.8 (C-4), 33.8 (C-3′), 36.5 (C-7‴), 42.1 (C-2″), 54.1 (C-2′), 55.3 (C-2), 62.3 (CH_2_CH_3_), 116.2 (C-3‴, H-5‴), 130.0 (C-1‴), 131.2 (C-2‴, C-6‴), 157.6 (C-4‴), 171.1 (C-1″), 173.6 (C-1′), 173.9 (C-1), 175.0 (C-5); HRESIMS (negative mode) *m*/*z* = 440.1487 [M − H]^−^ (calcd. for C_19_H_26_N_3_O_7_S, 440.1491).

### 3.4. Cell Culture

HT22 hippocampal neuronal cells were purchased from the Korean Cell Line Bank (Seoul, South Korea). R28 retinal cells were purchased from Kerafast (Kerafast, Inc., Boston, MA, USA), and BV2 microglial cells were obtained from Prof. Choon-Gon Jang, Sungkyunkwan University (Suwon, South Korea).

HT22 hippocampal neuronal cells and R28 retinal cells were cultured in Dulbecco’s modified Eagle’s minimum essential medium (DMEM) supplemented with penicillin (100 U/mL), streptomycin (100 µg/mL), and 10% fetal bovine serum (FBS) (Gibco) in a humidified atmosphere of 5% CO_2_ at 37 °C. Cells were cultured every two days using 0.05% trypsin.

BV2 microglial cells were cultured in DMEM mixed with penicillin (100 U/mL), streptomycin (100 µg/mL), and 10% FBS in a humidified atmosphere of 5% CO_2_ at 37 °C. Cells were cultured every two days using a pipette for detaching cells.

### 3.5. Sample Preparation

The 10 mg/mL DMSO stock solutions using the compounds were prepared and serially diluted 3-fold with DMSO. The amount and concentration of DMSO were always maintained in all conditions, regardless of the concentrations of the compounds of interest. Using a liquid handling station equipped with a pin tool system, we typically transferred 500 nL of the compound in DMSO solution into 100 µL of the medium, which resulted in a consistent DMSO concentration of 0.5%.

### 3.6. Cell Viability Assay

Cell viability in HT22 and R28 cells was evaluated using the MTT assay. The HT22 cells were seeded at a density of 3 × 10^3^ cells/well in a 96-well plate and R28 cells were plated at 10 × 10^3^ cells/well in a 96-well plate. After incubation for 24 h, compounds including NAC (1 mM) were added over 2 h, followed by the addition of 5 mM of glutamate for 22 h. A total of 50 µL of EZ-Cytox reagent (Daeil Lab) was added over 2 h. The plate was measured at 450 nm using a UV/VIS plate reader [36,37].

Cytotoxicity of compounds against the BV2 cells was determined using the MTT assay. The cells were added at the density of 3 × 10^4^ cells/well in a 96-well plate. After incubating for 24 h, the cells were treated with compounds and incubated for 24 h. After 24 h, 50 µL of media was removed and 50 µL of EZ-Cytox reagent was added over 1 h, and the plate was measured at 450 nm using a UV/VIS plate reader.

### 3.7. NO Production Assay

BV2 microglial cells were seeded at the density of 3 × 10^4^ cells/well in a 96-well plate. The cells were treated with compounds for 1 h before exposure of 1 µg/mL of LPS. After 24 h incubation with compound and LPS, nitrite in culture media was measured to assess NO production in BV2 cells using Griess reagent. In a 96-well plate, 50 µL of aliquots were mixed with 50 µL of Griess reagent (1% sulfanilamide, 0.1% naphtylethylenediamine dihydrochloride in 2% phosphoric acid) and incubated at room temperature for 15 min.; the plate was measured at 544 nm using a UV/VIS plate reader.

## 4. Conclusions

Three new compounds (**1**–**3**), together with 16 known compounds (**4**–**19**), were isolated and structurally characterized using chromatographic and spectroscopic methods. Among the compounds, phenolic compounds **4** and **5** were found to possess moderate neuroprotective activities in HT22 cells, while phenolic compounds **2**, **6**, and **7** exhibited weak activities in R28 cells. Compound **9** showed a moderate inhibitory effect on nitric oxide production in LPS-treated BV2 microglial cells.

Previous reports have revealed that phenolic compounds isolated from *G. elata* can protect neuronal cells through antioxidative, anti-inflammatory, anticonvulsive, neuroprotective, and circulatory system modulating effects, etc [8]. However, the investigation of other neuroprotective compounds needs to be evaluated to provide clarification for potential antagonistic or synergistic effects of multi-components contained in *G. elata*. Therefore, our investigation could provide additional information of neuroprotective compounds on hippocampal neuronal, retinal precursor, and microglial cells, which can contribute to the development of therapeutic agents for neuronal disease using phenolic compound mixtures derived from *G. elata*.

## Figures and Tables

**Table 1:** Nuclear magnetic resonance (NMR) spectroscopic data for compounds **1** and **2**.

Position	1 (CD_3_COCD_3_)		2 (CDCl_3_)	
	*δC*	*δ*_H_	*δ*_C_	*δ*_H_
1	129.2		131.6	
2	130.3	7.28, d (8.5)	129.9	6.99, d (8.5)
3	116.0	6.84, d (8.5)	115.6	6.62, d (8.5)
4	158.1		154.3	
5	116.0	6.84, d (8.5)	115.6	6.62, d (8.5)
6	130.3	7.28, d (8.5)	129.9	6.99, d (8.5)
7	70.4	4.94, s	35.6	4.41, s
1′	135.1		130.2	
2′	130.4	7.10, d (8.5)	131.1	7.08, d (1.5)
3′	115.6	6.89, d (8.5)	127.6	
4′	158.2		153.7	
5′	115.6	6.89, d (8.5)	115.8	6.67, d (8.0)
6′	130.4	7.10, d (8.5)	128.0	7.06, dd (8.0, 1.5)
7′	40.7	3.80, s	72.8	3.85,s
1″	133.5		65.9	3.57, q (7.0)
2″	130.5	7.02, d (8.0)	15.3	1.24, t (7.0)
3″	116.0	6.74, d (8.0)		
4″	156.5			
5″	116.0	6.74, d (8.0)		
6″	130.5	7.02, d (8.0)		

## Supplementary Materials

The following are available online at https://www.mdpi.com/2223-7747/9/8/1051/s1, S1: The ^1^H NMR spectral data of compounds **4**–**19**, Table S1: The screening of all isolated compounds for protective effects against HT22 cell death, Table S2: The screening of all isolated compounds (50 μM) for protective effects on R28 cell death, Table S3: The screening of all isolated compounds for inhibitory effects of nitric oxide production on LPS-treated BV2 cell lines. Figures S1–S8: The ^1^H, ^13^C, COSY, HSQC, and HMBC NMR, UV, IR, and HRESIMS spectrum of compound **1**, Figures S9–S16: The ^1^H, ^13^C, COSY, HSQC, and HMBC NMR, UV, IR, and HRESIMS spectrum of compound **2**, Figures S17–S24: The ^1^H, ^13^C, COSY, HSQC, and HMBC NMR, UV, IR, and HRESIMS spectrum of compound **3**.

## References

[1] Gooch C.L., Pracht E., Borenstein A.R. (2017). The burden of neurological disease in the United States: A summary report and call to action. Ann. Neurol..

[2] Kang Y., Tiziani S., Park G., Kaul M., Paternostro G. (2014). Cellular protection using Flt3 and PI3Kα inhibitors demonstrates multiple mechanisms of oxidative glutamate toxicity. Nat. Commun..

[3] Dixon S.J., Lemberg K.M., Lamprecht M.R., Skouta R., Zaitsev E.M., Gleason C.E., Patel D.N., Bauer A.J., Cantley A.M., Yang W.S. (2012). Ferroptosis: An iron-dependent form of nonapoptotic cell death. Cell.

[4] Wässle H. (2004). Parallel processing in the mammalian retina. Nat. Rev. Neurosci..

[5] Yu D.-Y., Cringle S.J. (2001). Oxygen distribution and consumption within the retina in vascularised and avascular retinas and in animal models of retinal disease. Prog. Retin. Eye Res..

[6] London A., Benhar I., Schwartz M. (2013). The retina as a window to the brain—From eye research to CNS disorders. Nat. Rev. Neurol..

[7] Kirkley K.S., Popichak K.A., Afzali M.F., Legare M.E., Tjalkens R.B. (2017). Microglia amplify inflammatory activation of astrocytes in manganese neurotoxicity. J. Neuroinflamm..

[8] Zhan H.-D., Zhou H.-Y., Sui Y.-P., Du X.-L., Wang W.-h., Dai L., Sui F., Huo H.-R., Jiang T.-L. (2016). The rhizome of *Gastrodia elata* blume—An ethnopharmacological review. J. Ethnopharmacol..

[9] Ojemann L.M., Nelson W.L., Shin D.S., Rowe A.O., Buchanan R.A. (2006). Tian ma, an ancient Chinese herb, offers new options for the treatment of epilepsy and other conditions. Epilepsy Behav..

[10] Lee J.Y., Jang Y.W., Kang H.S., Moon H., Sim S.S., Kim C.J. (2006). Anti-inflammatory action of phenolic compounds from *Gastrodia elata* root. Arch. Pharm. Res..

[11] Huang Z.-B., Wu Z., Chen F.-K., Zou L.-B. (2006). The protective effects of phenolic constituents from *Gastrodia elata* on the cytotoxicity induced by KCl and glutamate. Arch. Pharm. Res..

[12] Jung T.Y., Suh S.I., Lee H., Kim I.S., Kim H.J., Yoo H.S., Lee S.R. (2007). Protective effects of several components of *Gastrodia elata* on lipid peroxidation in gerbil brain homogenates. Phytother. Res..

[13] Jang J.-H., Son Y., Kang S.S., Bae C.-S., Kim J.-C., Kim S.-H., Shin T., Moon C. (2015). Neuropharmacological potential of *Gastrodia elata* Blume and its components. Evid. Based Complement. Alternat. Med..

[14] Wang Y., Lin S., Chen M., Jiang B., Guo Q., Zhu C., Wang S., Yang Y., Shi J. (2012). Chemical constituents from aqueous extract of *Gastrodia elata*. China J. Chin. Mater. Med..

[15] Tang C., Wang L., Liu X., Cheng M., Xiao H. (2016). Chemical fingerprint and metabolic profile analysis of ethyl acetate fraction of *Gastrodia elata* by ultra performance liquid chromatography/quadrupole-time of flight mass spectrometry. J. Chromatogr. B.

[16] Li N., Wang K.-J., Chen J.-J., Zhou J. (2007). Phenolic compounds from the rhizomes of *Gastrodia elata*. J. Asian Nat. Prod. Res..

[17] Han A.R., Ji Shin H., Rim Jeon H., Lee J.H., Lee D., Seo E.K. (2011). Two new phenolic compounds from the rhizomes of *Gastrodia elata* Blume. Helv. Chim. Acta.

[18] Taguchi H., Yosioka I., Yamasaki K., Kim I.H. (1981). Studies on the constituents of *Gastrodia elata* Blume. Chem. Pharm. Bull..

[19] Yang X.-D., Zhu J., Yang R., Liu J.-P., Li L., Zhang H.-B. (2007). Phenolic constituents from the rhizomes of *Gastrodia elata*. Nat. Prod. Res..

[20] Huang Z.-B., Song D.-M., Chen F.-K. (2005). The chemical constituents isolated from *Gastrodia elata* BL. Chin. J. Med. Chem..

[21] Ciuffreda P., Casati S., Manzocchi A. (2007). Complete ^1^H and ^13^C NMR spectral assignment of *α*-and *β*-adenosine, 2′-deoxyadenosine and their acetate derivatives. Magn. Reson. Chem..

[22] Guo Q.-L., Wang Y.-N., Zhu C.-G., Chen M.-H., Jiang Z.-B., Chen N.-H., Song X.-Y., Zhang M.-J., Shi J.-G. (2015). 4-Hydroxybenzyl-substituted glutathione derivatives from *Gastrodia elata*. J. Asian Nat. Prod. Res..

[23] Guan P., Shi J.-M., Gao Y.-Q. (2008). Study on the volatile components of *Gastrodia elata*. J. Sichuan Norm. Univ. Nat. Sci..

[24] Tan S., Sagara Y., Liu Y., Maher P., Schubert D. (1998). The regulation of reactive oxygen species production during programmed cell death. J. Cell Biol..

[25] Seigel G.M. (2014). R28 retinal precursor cells: The first 20 years. Mol. Vis..

[26] Boyce J.A. (2008). Eicosanoids in asthma, allergic inflammation, and host defense. Curr. Mol. Med..

[27] Yuan L., Qian L., Qian Y., Liu J., Yang K., Huang Y., Wang C., Li Y., Mu X. (2019). Bisphenol F-induced nurotoxicity toward zebrafish embryos. Environ. Sci. Technol..

[28] Kubo T., Maezawa N., Osada M., Katsumura S., Funae Y., Imaoka S. (2004). Bisphenol A, an environmental endocrine-disrupting chemical, inhibits hypoxic response via degradation of hypoxia-inducible factor 1α (HIF-1α): Structural requirement of bisphenol A for degradation of HIF-1α. Biochem. Biophys. Res. Commun..

[29] Jiang G., Wu H., Hu Y., Li J., Li Q. (2014). Gastrodin inhibits glutamate-induced apoptosis of PC12 cells via inhibition of CaMKII/ASK-1/p38 MAPK/p53 signaling cascade. Cell. Mol. Neurobiol..

[30] Oliveira M.R., Brasil F.B., Fürstenau C.R. (2019). Nrf2 mediates the anti-apoptotic and anti-inflammatory effects induced by gastrodin in hydrogen peroxide–treated SH-SY5Y cells. J. Mol. Neurosci..

[31] Xu X., Lu Y., Bie X. (2007). Protective effects of gastrodin on hypoxia-induced toxicity in primary cultures of rat cortical neurons. Planta Med..

[32] Qiu C.-W., Liu Z.-Y., Zhang F.-L., Zhang L., Li F., Liu S.-Y., He J.-Y., Xiao Z.-C. (2019). Post-stroke gastrodin treatment ameliorates ischemic injury and increases neurogenesis and restores the Wnt/β-Catenin signaling in focal cerebral ischemia in mice. Brain Res..

[33] Dai J.-N., Zong Y., Zhong L.-M., Li Y.-M., Zhang W., Bian L.-G., Ai Q.-L. (2011). Gastrodin inhibits expression of inducible NO synthase, cyclooxygenase-2 and proinflammatory cytokines in cultured LPS-stimulated microglia via MAPK pathways. PLoS ONE.

[34] Ha J.-H., Lee D.-U., Lee J.-T., Kim J.-S., Yong C.-S., Kim J.-A., Ha J.-S. (2000). 4-Hydroxybenzaldehyde from *Gastrodia elata* B1. is active in the antioxidation and GABAergic neuromodulation of the rat brain. J. Ethnopharmacol..

[35] Lee Y.S., Ha J.-H., Yong C.S., Lee D.-U., Huh K., Kang Y.S., Lee S.H., Jung M.-W., Kim J.-A. (1999). Inhibitory effects of constituents of *Gastrodia elata* Bl. on glutamate-induced apoptosis in IMR-32 human neuroblastoma cells. Arch. Pharm. Res..

[36] Fukui M., Song J.-H., Choi J., Choi H.J., Zhu B.T. (2009). Mechanism of glutamate-induced neurotoxicity in HT22 mouse hippocampal cells. Eur. J. Pharmacol..

[37] Kim K.-A., Kang S.W., Ahn H.R., Song Y., Yang S.J., Jung S.H. (2015). Leaves of persimmon (*Diospyros kaki* Thunb.) ameliorate *N*-methyl-*N*-nitrosourea (MNU)-induced retinal degeneration in mice. J. Agric. Food Chem..

