# Supplementary material for: Constituents of Gastrodia elata and Their Neuroprotective Effects in HT22 Hippocampal Neuronal, R28 Retinal Cells, and BV2 Microglial Cells
Source: Plants (Basel). 2020 Aug 18;9(8):1051. doi: 10.3390/plants9081051 (PMC7465223; doi:10.3390/plants9081051)
Supplement: Supplementary file 1 [file plants-09-01051-s001.pdf]

plants

Article

Constituents of *Gastrodia elata* and Their Neuroprotective Effects in HT22 Hippocampal Neuronal, R28 Retinal Cells, and BV2 Microglial Cells

Hye Mi Kim [1,†], Jaeyoung Kwon [2,†], Kyerim Lee [2,3], Jae Wook Lee [2], Dae Sik Jang [1,3,*] and Hak Cheol Kwon [2,*]

[1] College of Pharmacy, Kyung Hee University, Seoul 02447, Republic of Korea; hyemi586@gmail.com (H.M.K.); dsjang@khu.ac.kr (D.S.J.)
[2] KIST Gangneung Institute of Natural Products, Korea Institute of Science and Technology (KIST), Gangneung 25451, Republic of Korea; kjy1207@kist.re.kr (J.K.); klim8@kist.re.kr (K.L.); jwlee5@kist.re.kr (J.W.L.); hkwon@kist.re.kr (H.C.K.)
[3] KHU-KIST Department of Converging Science and Technology, Kyung Hee University, Seoul 02447, Republic of Korea; klim8@kist.re.kr (K.L.); dsjang@khu.ac.kr (D.S.J.)
* Correspondence: hkwon@kist.re.kr (H.C.K.); dsjang@khu.ac.kr (D.S.J.); /
† These authors contributed equally to this work and joint first authors

List of Supporting Information

S1. ^{1}H and ^{13}C NMR spectral data of compounds **4–19**.
Table S1. Screening of all isolated compounds for protective effects against HT22 cell death caused by glutamate-induced toxicity.
Table S2. Screening of all isolated compounds (50 μM) for protective effects on R28 cell death caused by H_2O_2 induced toxicity.
Table S3. Screening of all isolated compounds for inhibitory effects of nitric oxide production on LPS treated BV2 cell lines.
Figure S1. The ^{1}H NMR spectrum of compound **1** (500 MHz, CD_3COCD_3).
Figure S2. The ^{13}C NMR spectrum of compound **1** (125 MHz, CD_3COCD_3).
Figure S3. The ^{1}H-^{1}H COSY NMR spectrum of compound **1** (500MHz, CD_3COCD_3).
Figure S4. The HSQC NMR spectrum of compound **1** (500 MHz, CD_3COCD_3).
Figure S5. The HMBC NMR spectrum of compound **1** (500 MHz, CD_3COCD_3).
Figure S6. The UV spectrum of compound **1** (CH_3OH).
Figure S7. The IR spectrum of compound **1**.
Figure S8. The HRESIMS spectrum of compound **1**.
Figure S9. The ^{1}H NMR spectrum of compound **2** (500 MHz, $CDCl_3$).
Figure S10. The ^{13}C NMR spectrum of compound **2** (125 MHz, $CDCl_3$).
Figure S11. The ^{1}H-^{1}H COSY NMR spectrum of compound **2** (500 MHz, $CDCl_3$).
Figure S12. The HSQC NMR spectrum of compound **2** (500 MHz, $CDCl_3$).
Figure S13. The HMBC NMR spectrum of compound **2** (500 MHz, $CDCl_3$).
Figure S14. The UV spectrum of compound **2** (CH_3OH).
Figure S15. The IR spectrum of compound **2**.
Figure S16. The HRESIMS spectrum of compound **2**.
Figure S17. The ^{1}H NMR spectrum of compound **3** (500 MHz, CD_3OD).
Figure S18. The ^{13}C NMR spectrum of compound **3** (125 MHz, CD_3OD).
Figure S19. The ^{1}H-^{1}H COSY NMR spectrum of compound **3** (500 MHz, CD_3OD).
Figure S20. The HSQC NMR spectrum of compound **3** (500 MHz, CD_3OD).
Figure S21. The HMBC NMR spectrum of compound **3** (500 MHz, CD_3OD).
Figure S22. The UV spectrum of compound **3** (CH_3OH).
Figure S23. The IR spectrum of compound **3**.
Figure S24. The HRESIMS spectrum of compound **3**.

S1. ^{1}H and ^{13}C NMR spectral data of compounds **4–19**.

4-[[4-(ethoxymethyl)phenoxy]methyl]phenol (**4**) exhibited following data: white amorphous powder; ^{1}H NMR (500 MHz, CD_3OD) δ : 7.26 (2H, d, *J* = 8.0 Hz, H-2, H-6), 7.26 (2H, d, *J* = 8.0 Hz, H-2′, H-6′), 6.92 (2H, d, *J* = 8.0 Hz, H-3′, H-5′), 6.76 (2H, d, *J* = 8.0 Hz, H-3, H-5), 4.93 (2H, s, H_2-7), 4.44 (2H, s, H_2-7′), 3.53 (2H, q, *J* = 7.0 Hz, $\underline{CH_2}CH_3$), 1.23 (3H, t, *J* = 7.0 Hz, $CH_2\underline{CH_3}$);; ^{13}C NMR (125 MHz, CD_3OD) δ : 129.0 (C-1), 129.7 (C-2, 6), 115.6 (C-3, C-5), 155.9 (C-4), 70.0 (C-7), 130.8 (C-1′), 129.6 (C-2′, C-6′), 115.0 (C-3′, C-5′), 158.6 (C-4′), 72.6 (C-7′), 65.7 ($\underline{CH_2}CH_3$), 15.3 ($CH_2\underline{CH_3}$).

Gastrol A (**5**) exhibited following data: white amorphous powder; ^{1}H NMR (500 MHz, CD_3COCD_3) δ : 7.34 (2H, d, *J* = 8.5 Hz, H-2″, 6″), 7.31 (2H, d, *J* = 8.5 Hz, H-2, H-6), 7.23 (2H, d, *J* = 8.5 Hz, H-2′, H-6′), 7.01 (2H, d, *J* = 8.5 Hz, H-3, H-5), 6.89 (2H, d, *J* = 8.5 Hz, H-3″, H-5″), 6.85 (2H, d, *J* = 8.5 Hz, H-3′, H-5′), 5.03 (2H, s, H_2-7″), 4.48 (2H, s, H_2-7), 4.46 (2H, s, H_2-7′); ^{13}C NMR (125 MHz, CD_3COCD_3) δ : 132.0 (C-1), 130.2 (C-2, C-6), 115.5 (C-3, C-5), 159.5 (C-4), 72.0 (C-7), 130.6 (C-1′), 130.4 (C-2′, C-6′), 115.9 (C-3′, C-5′), 157.8 (C-4′), 72.3 (C-7′), 129.2 (C-1″), 130.4 (C-2″, C-6″), 116.1 (C-3″, 5″), 158.2 (C-4″), 70.5 (C-7″).

Bis(4-hydroxyphenyl)methane (**6**) exhibited following data: white amorphous powder; ^{1}H NMR (500 MHz, CD_3OD) δ : 6.96 (4H, d, *J* = 8.5 Hz, H-2, H-6, H-2′, H-6′), 6.68 (4H, d, *J* = 8.5 Hz, H-3, H-5, H-3′, H-5′), 3.74 (2H, s, H_2-7); ^{13}C NMR (125 MHz, CD_3OD) δ : 134.4 (C-1, C-1′), 130.9 (C-2, C-6, C-2′, C-6′), 116.2 (C-3, C-5, C-3′, C-5′), 156.6 (C-4, C-4′), 41.3 (C-7).

4-Hydroxybenzyl vanillyl ether (**7**) exhibited following data: white amorphous powder; ^{1}H NMR (500 MHz, CD_3OD) δ : 7.17 (2H, d, *J* = 8.5 Hz, H-2, H-6), 6.91 (1H, d, *J* = 1.5 Hz, H-2′), 6.77 (1H, dd, *J* = 8.0, 1.5 Hz, H-6′), 6.77 (1H, d, *J* = 8.0 Hz, H-5′), 6.76 (2H, d, *J* = 8.5 Hz, H-3, H-5), 4.41 (2H, s, H_2-7′), 4.41 (2H, s, H_2-7), 3.84 (3H, s, 3′-OCH_3); ^{13}C NMR (125 MHz, CD_3OD) δ : 131.2 (C-1), 131.1 (C-2, 6), 116.3 (C-3, 5), 158.5 (C-4), 72.9 (C-7), 130.5 (C-1′), 113.2 (C-2′), 149.2 (C-3′), 147.6 (C-4′), 116.1 (C-5′), 122.5 (C-6′), 73.1 (C-7′), 56.6 (3′-OCH_3).

Bis(4-hydroxybenzyl)ether (**8**) exhibited following data: colorless oil; ^{1}H NMR (500 MHz, CD_3OD) δ : 7.16 (4H, d, *J* = 8.5 Hz, H-2, H-6, H-2′, H-6′), 6.76 (4H, d, *J* = 8.5 Hz, H-3, H-5, H-3′, H-5′), 4.40 (4H, s, H_2-7, H_2-7′); ^{13}C NMR (125 MHz, CD_3OD) δ : 130.5 (C-1, C-1′), 131.1 (C-2, C-6, C-2′, C-6′), 116.3 (C-3, C-5, C-3′, C-5′), 158.7 (C-4, C-4′), 72.9 (C-7, C-7′).

2,4-Bis(4-hydroxybenzyl)phenol (**9**) exhibited following data: brownish amorphous powder; ^{1}H NMR (500 MHz, CD_3OD) δ : 6.99 (2H, d, *J* = 8.5 Hz, H-2′, H-6′), 6.92 (2H, d, *J* = 8.5 Hz, H-2″, H-6″), 6.80 (1H, d, *J* = 2.0 Hz, H-2), 6.78 (1H, dd, *J* = 8.0, 2.0 Hz, H-5), 6.66 (1H, d, *J* = 8.0 Hz, H-6), 6.66 (2H, d, *J* = 8.5 Hz, H-3′, H-5′), 6.66 (2H, d, *J* = 8.5 Hz, H-3″, H-5″), 3.78 (2H, s, H_2-7′), 3.69 (2H, s, H_2-7″); ^{13}C NMR (125 MHz, CD_3OD) δ : 154.4 (C-1), 133.9 (C-2), 132.1 (C-3), 134.6 (C-4), 128.4 (C-5), 116.1 (C-6), 129.7 (C-1′), 131.0 (C-2′, C-6′), 116.2 (C-3′, C-5′), 156.4 (C-4′), 35.9 (C-7′), 134.3 (C-1″), 130.8 (C-2″, C-6″), 116.1 (C-3″, C-5″), 156.5 (C-4″), 41.4 (C-7″).

Gastrodigenin (**10**) exhibited following data: white amorphous powder; ^{1}H NMR (500 MHz, CD_3OD) δ : 7.16 (2H, d, *J* = 8.5 Hz, H-2, H-6), 6.76 (2H, d, *J* = 8.5 Hz, H-3, H-5), 4.39 (2H, s, H_2-7); ^{13}C NMR (125 MHz, CD_3OD) δ : 130.4 (C-1), 131.1 (C-2, C-6), 116.3 (C-3, C-5), 158.5 (C-4), 72.8 (C-7).

4-Hydroxybenzyl ethyl ether (**11**) exhibited following data: white amorphous powder; ^{1}H NMR (500 MHz, $CDCl_3$) δ : 7.10 (2H, d, *J* = 8.0 Hz, H-2, H-6), 6.72 (2H, d, *J* = 8.0 Hz, H-3, H-5), 4.42 (2H, s, H_2-7), 3.53 (2H, q, *J* = 7.0 Hz, $\underline{CH_2}CH_3$), 1.22 (3H, t, *J* = 7.0 Hz, $CH_2\underline{CH_3}$); ^{13}C NMR (125 MHz, $CDCl_3$) δ : 130.2 (C-1), 129.9 (C-2, C-6), 115.5 (C-3, C-5), 155.7 (C-4), 72.7 (C-7), 65.7 ($\underline{CH_2}CH_3$), 15.3 ($CH_2\underline{CH_3}$).

Gastrodin (**12**) exhibited following data: white amorphous powder; ^{1}H NMR (500 MHz, CD_3OD) δ : 7.28 (2H, d, *J* = 8.5 Hz, H-2, H-6), 7.08 (2H, d, *J* = 8.5 Hz, H-3, H-5), 4.89 (1H, d, *J* = 7.5 Hz, Glc H-1), 4.54 (2H, s, H_2-7), 3.89 (1H, dd, *J* = 12.0, 2.0 Hz, Glc H_2-6a), 3.70 (1H, dd, *J* = 12.0, 5.0 Hz, Glc H_2-6b), 3.46-3.37 (4H, Glc H-2, H-3, H-4, H-5); ^{13}C NMR (125 MHz, CD_3OD) δ : 136.8 (C-1), 129.6 (C-2, C-6), 117.8 (C-3, C-5), 158.7 (C-4), 65.0 (C-7), 102.6 (Glc C-1), 75.1 (Glc C-2), 78.3 (Glc C-3), 71.6 (Glc C-4), 78.2 (Glc C-5), 62.7 (Glc C-6).

4-Hydroxybenzaldehyde (**13**) exhibited following data: brownish amorphous powder; ^{1}H NMR (500 MHz, CD_3OD) δ : 9.76 (1H, s, $C\underline{H}O$) 6.96 (2H, d, *J* = 8.5 Hz, H-2, H-6), 6.68 (2H, d, *J* = 8.5 Hz, H-3, H-5); ^{13}C NMR (125 MHz, CD_3OD) δ : 130.4 (C-1), 133.6 (C-2, C-6), 117.1 (C-3, C-5), 165.5 (C-4), 193.0 ($\underline{C}HO$).

3,5-Dimethoxybenzoic acid-4-*O*-*β*-D-glucopyranoside (**14**) exhibited following data: white amorphous powder; ^{1}H NMR (500 MHz, DMSO-d_6) δ : 7.22 (2H, s, H-2, H-6), 5.11 (1H, d, *J* = 7.0 Hz,

Glc H-1), 3.59-3.06 (6H, Glc H-2, H-3, H-4, H-5, H_2-6), 3.80 (6H, s, 3-OCH_3, 5-OCH_3); ^{13}C NMR (125 MHz, DMSO-d_6) δ : 136.0 (C-1), 116.9 (C-2, C-6), 161.9 (C-3, C-5), 147.6 (C-4), 176.8 ($\underline{C}$OOH), 111.6 (Glc C-1), 83.9 (Glc C-2), 87.1 (Glc C-3), 79.6 (Glc C-4), 86.3 (Glc C-5), 70.5 (Glc C-6), 66.0 (3-OCH_3, 5-OCH_3).

Parishin E (**15**) exhibited following data: yellowish oil; ^{1}H NMR (500 MHz, CD_3OD) δ : 7.31 (2H, d, *J* = 8.5 Hz, H-2′, H-6′), 7.08 (2H, d, *J* = 8.5 Hz, H-3′, H-5′), 5.06 (2H, d, *J* = 2.5 Hz, H_2-7′), 4.91 (1H, d, *J* = 7.5 Hz, Glc H-1), 3.89 (1H, dd, *J* = 12.0, 2.0 Hz, Glc H_2-6a), 3.70 (1H, dd, *J* = 12.0, 5.5 Hz, Glc H_2-6b), 3.47-3.30 (4H, Glc, H-2, H-3, H-4, H-5), 2.96 (1H, d, *J* = 15.5 Hz, H_2-3a), 2.92 (1H, d, *J* = 16.0 Hz, H_2-1a), 2.85 (1H, d, *J* = 15.5 Hz, H_2-3b), 2.79 (1H, d, *J* = 16.0 Hz, H_2-1b); ^{13}C NMR (125 MHz, CD_3OD) δ : 44.2 (C-1), 75.1 (C-2), 44.5 (C-3), 131.4 (C-1′), 131.0 (C-2′, C-6′), 117.9 (C-3′, C-5′), 159.2 (C-4′), 67.4 (C-7′), 102.4 (Glc C-1), 75.1 (Glc C-2), 78.3 (Glc C-3), 71.6 (Glc C-4), 78.2 (Glc C-5), 62.7 (Glc C-6), 171.5 ($\underline{C}$OOR), 173.9 (COOH), 177.1 (COOH).

Adenosine (**16**) exhibited following data: white amorphous powder; ^{1}H NMR (500 MHz, DMSO-d_6) δ : 8.35 (1H, s, H-8), 8.14 (1H, s, H-2), 7.37 (2H, br s, $N\underline{H_2}$), 5.88 (1H, d, *J* = 6.0 Hz, H-1′), 5.46 (1H, br d, *J* = 6.0 Hz, 2′-OH), 5.45 (1H, br dd, *J* = 7.5, 4.5 Hz, 5′-OH), 5.20 (1H, br d, *J* = 4.5 Hz, 3′-OH), 4.61 (1H, ddd, *J* = 6.0, 6.0, 5.0 Hz, H-2′), 4.14 (1H, ddd, *J* = 5.0, 4.5, 3.0 Hz, H-3′), 3.96 (1H, ddd, *J* = 3.5, 3.5, 3.0 Hz, H-4′), 3.67 (1H, ddd, *J* = 12.0, 4.5, 3.5 Hz, H_2-5′a), 3.55 (1H, ddd, *J* = 12.0, 7.5, 3.5 Hz, H_2-5′b); ^{13}C NMR (125 MHz, DMSO-d_6) δ : 152.3 (C-2), 149.0 (C-4), 119.3 (C-5), 156.1 (C-6), 139.9 (C-8), 87.9 (C-1′), 73.4 (C-2′), 70.6 (C-3′), 85.8 (C-4′), 61.6 (C-5′).

S-(4-Hydroxybenzyl) glutathione (**17**) exhibited following data: white amorphous powder; ^{1}H NMR (500 MHz, DMSO-d_6) δ : 7.09 (2H, d, *J* = 8.5 Hz, H-2‴, H-6‴), 6.69 (2H, d, *J* = 8.5 Hz, H-3‴, H-5‴), 4.50 (1H, dd, *J* = 9.0, 5.0 Hz, H-2′), 3.71 (1H, d, *J* = 2.5 Hz, H_2-2″a), 3.63 (1H, d, *J* = 2.5 Hz, H_2-2″b), 3.63 (2H, s, H_2-7‴), 3.40 (1H, t, *J* = 6.5 Hz, H-2), 2.78 (1H, dd, *J* = 14.0, 5.0 Hz, H_2-3′a), 2.55 (1H, dd, *J* = 14.0, 9.0 Hz, H_2-3′b), 2.32 (2H, m, H_2-4), 1.93 (1H, br dt, *J* = 6.5, 6.5 Hz, H-3); ^{13}C NMR (125 MHz, DMSO-d_6) δ : 171.7 (C-1), 53.1 (C-2), 26.8 (C-3), 31.5 (C-4), 170.9 (C-5), 170.7 (C-1′), 52.2 (C-2′), 33.0 (C-3′), 170.6 (C-1″), 41.2 (C-2″), 128.1 (C-1‴), 129.9 (C-2‴, C-6‴), 115.1 (C-3‴, C-5‴), 156.3 (C-4‴), 34.8 (C-7‴).

Palmitic acid ethyl ester (**18**) exhibited following data: yellowish waxy-like; ^{1}H NMR (500 MHz, $CDCl_3$) δ : 4.10 (2H, q, *J* = 7.0 Hz, $\underline{CH_2}CH_3$), 2.26 (2H, t, *J* = 7.5 Hz, H_2-2), 1.60 (2H, m, H_2-3), 1.28-1.22 (24H, br m, H_2-4, H_2-5, H_2-6, H_2-7, H_2-8, H_2-9, H_2-10, H_2-11, H_2-12, H_2-13, H_2-14, H_2-15) 1.23 (3H, t, *J* = 7.0 Hz, $CH_2\underline{CH_3}$), 0.86 (3H, t, *J* = 7.0 Hz, H_3-16); ^{13}C NMR (125 MHz, $CDCl_3$) δ : 174.2 (C-1), 34.6 (C-2), 25.2 (C-3), 29.4 (C-4), 29.6 (C-5), 30.0, 30.0, 29.9, 29.9, 29.9, 29.8, 29.7 (C-6, C-7, C-8, C-9, C-10, C-11, C-12), 29.5 (C-13), 32.2 (C-14), 22.9 (C-15), 14.3 (C-16), 60.4 ($\underline{CH_2}CH_3$), 14.5 ($CH_2\underline{CH_3}$).

Linoleic acid ethyl ester (**19**) exhibited following data: colorless oil; ^{1}H NMR (500 MHz, $CDCl_3$) δ : 5.33 (4H, m, H-9, H-10, H-12, H-13), 4.13 (2H, q, *J* = 7.0 Hz, $\underline{CH_2}CH_3$), 2.75 (2H, t, *J* = 6.5 Hz, H_2-11), 2.26 (2H, t, *J* = 7.5 Hz, H_2-2), 2.02 (4H, br dt, *J* = 7.0, 7.0 Hz, H_2-8, H_2-14), 1.60 (2H, br m, H-3), 1.36-1-29 (14H, br m, H_2-4, H_2-5, H_2-6, H_2-7, H_2-15, H_2-16, H_2-17), 1.23 (3H, t, *J* = 7.0 Hz, $CH_2\underline{CH_3}$), 0.87 (3H, t, *J* = 7.0 Hz, H_3-18); ^{13}C NMR (125 MHz, $CDCl_3$) δ : 174.1 (C-1), 34.6 (C-2), 31.7 (C-3), 29.8, 29.6, 29.4, 29.3, 29.3, 25.2, 22.8 (C-4, C-5, C-6, C-7, C-15, C-16, C-17), 27.4 (C-8), 128.1 (C-9), 130.4 (C-10), 25.8 (C-11), 130.3 (C-12), 128.2 (C-13), 27.4 (C-14), 14.3 (C-18), 60.4 ($\underline{CH_2}CH_3$), 14.5 ($CH_2\underline{CH_3}$).

Table S1. Screening of all isolated compounds for protective effects against HT22 cell death caused by glutamate-induced toxicity.

Compound	Concentration (μM)	Cell viability (%)	Compound	Concentration (μM)	Cell viability (%)
	5.6	38.27 ± 5.96		5.6	22.14 ± 2.82
**1**	16.6	33.62 ± 3.90	**2**	16.6	22.88 ± 3.46
	50.0	71.48 ± 6.68		50.0	72.26 ± 7.41
	5.6	37.94 ± 3.77		5.6	120.02 ± 2.41
**3**	16.6	51.75 ± 3.28	**4**	16.6	95.83 ± 14.01
	50.0	25.20 ± 2.20		50.0	53.17 ± 0.12
	5.6	110.34 ± 2.78		5.6	22.51 ± 1.90
**5**	16.6	110.34 ± 2.71	**6**	16.6	25.71 ± 2.71
	50.0	98.02 ± 0.78		50.0	26.28 ± 9.32
	5.6	24.33 ± 4.86		5.6	32.68 ± 1.32
**7**	16.6	32.69 ± 2.03	**8**	16.6	32.47 ± 3.60
	50.0	85.61 ± 10.78		50.0	100.35 ± 1.49
	5.6	31.79 ± 9.05		5.6	22.72 ± 1.82
**9**	16.6	20.67 ± 4.07	**10**	16.6	19.13 ± 3.92
	50.0	17.37 ± 2.18		50.0	23.79 ± 0.19
	5.6	30.78 ± 4.96		5.6	32.42 ± 1.45
**11**	16.6	27.67 ± 9.04	**12**	16.6	28.60 ± 2.89
	50.0	24.90 ± 3.26		50.0	23.07 ± 1.12
	5.6	21.75 ± 8.62		5.6	22.86 ± 10.6
**13**	16.6	20.21 ± 0.72	**14**	16.6	24.10 ± 3.53
	50.0	20.07 ± 0.43		50.0	20.16 ± 1.47
	5.6	34.32 ± 3.65		5.6	37.70 ± 0.96
**15**	16.6	33.15 ± 3.21	**16**	16.6	59.00 ± 6.08
	50.0	23.59 ± 0.90		50.0	76.25 ± 5.31
	5.6	33.95 ± 6.46		5.6	45.92 ± 3.18
**17**	16.6	54.24 ± 18.69	**18**	16.6	57.65 ± 4.64
	50.0	27.75 ± 1.21		50.0	50.30 ± 6.46
	5.6	30.32 ± 6.53	**DMSO**	-	100.00 ± 2.00
**19**	16.6	31.50 ± 6.60	**Glu**	5 mM	44.62 ± 2.00
	50.0	19.65 ± 1.92	**NAC**	1 mM	102.40 ± 0.95

Table S2. Screening of all isolated compounds (50 μM) for protective effects on R28 cell death caused by H_2O_2 induced toxicity.

No	Cell viability (%)	No	Cell viability (%)	No	Cell viability (%)
**1**	7.99 ± 0.13	**2**	79.88 ± 0.59	**3**	53.47 ± 3.14
**4**	31.60 ± 3.47	**5**	55.27 ± 0.18	**6**	76.13 ± 0.04
**7**	82.66 ± 2.49	**8**	40.88 ± 4.19	**9**	19.75 ± 9.86
**10**	53.07 ± 2.87	**11**	49.41 ± 9.92	**12**	52.01 ± 2.52
**13**	54.10 ± 1.33	**14**	53.99 ± 6.32	**15**	55.90 ± 2.22
**16**	58.30 ± 3.37	**17**	57.64 ± 4.14	**18**	54.49 ± 5.13
**19**	12.91 ± 4.62	**DMSO**	100.00 ± 3.22	**Glu (5 mM)**	50.77 ± 0.02
**NAC (1 mM)**	96.70 ± 0.95				

Table S3. Screening of all isolated compounds for inhibitory effects of nitric oxide production on LPS treated BV2 cell lines.

Compound	Concentration (μM)	Litrite (μM)	Compound	Concentration (μM)	Litrite[a] (μM)
**1**	0.2	27.91 ± 3.09	**2**	0.2	30.80 ± 0.33
	1.8	27.52 ± 1.66		1.8	25.88 ± 0.22
	16.6	29.47 ± 2.21		16.6	27.91 ± 0.44
	50.0	25.17 ± 3.65		50.0	28.77 ± 0.11
**3**	0.2	28.84 ± 0.66	**4**	0.2	28.30 ± 1.21
	1.8	27.13 ± 0.66		1.8	27.91 ± 0.88
	16.6	26.73 ± 0.99		16.6	23.53 ± 0.00
	50.0	25.48 ± 0.77		50.0	20.09 ± 1.10
**5**	0.2	26.34 ± 1.55	**6**	0.2	29.86 ± 0.99
	1.8	25.88 ± 2.65		1.8	23.77 ± 0.11
	16.6	25.56 ± 0.66		16.6	25.95 ± 0.11
	50.0	23.38 ± 1.10		50.0	30.02 ± 0.11
**7**	0.2	28.92 ± 0.77	**8**	0.2	28.76 ± 1.21
	1.8	25.41 ± 1.33		1.8	27.36 ± 0.11
	16.6	26.97 ± 0.44		16.6	27.67 ± 0.33
	50.0	26.03 ± 1.10		50.0	28.06 ± 1.99
**9**	0.2	27.52 ± 0.33	**10**	0.2	27.91 ± 1.32
	1.8	29.08 ± 0.33		1.8	25.95 ± 1.43
	16.6	27.98 ± 2.10		16.6	26.97 ± 0.22
	50.0	9.94 ± 2.78		50.0	27.36 ± 3.87
**11**	0.2	29.16 ± 1.33	**12**	0.2	27.83 ± 0.77
	1.8	22.98 ± 0.11		1.8	27.20 ± 1.44
	16.6	23.92 ± 0.99		16.6	26.66 ± 0.88
	50.0	26.11 ± 1.44		50.0	25.95 ± 0.99
**13**	0.2	30.64 ± 0.55	**14**	0.2	27.44 ± 0.44
	1.8	22.59 ± 0.22		1.8	24.55 ± 1.88
	16.6	22.83 ± 0.11		16.6	23.84 ± 1.10
	50.0	26.81 ± 0.00		50.0	23.22 ± 0.22
**15**	0.2	27.98 ± 0.77	**16**	0.2	29.47 ± 2.21
	1.8	27.83 ± 0.55		1.8	26.73 ± 0.77
	16.6	24.39 ± 1.21		16.6	25.95 ± 2.10
	50.0	26.81 ± 0.88		50.0	24.47 ± 1.77
**17**	0.2	32.20 ± 0.11	**18**	0.2	32.13 ± 2.65
	1.8	29.10 ± 2.65		1.8	27.91 ± 1.10
	16.6	26.11 ± 2.32		16.6	26.19 ± 0.66
	50.0	25.56 ± 1.77		50.0	26.03 ± 0.88
**19**	0.2	34.16 ± 1.44	**DMSO**	-	2.91 ± 0.22
	1.8	29.08 ± 2.54			
	16.6	26.97 ± 2.65	**LPS**	1 μg/ml	32.43 ± 0.44
	50.0	25.95 ± 1.44			

[1] Secreted nitric oxide levels were determined by Griess reagent. [2] 1 μg/mL of LPS was used in NO production and cell viability assay. The % values are representative relative cell viabilities compared with DMSO treated cell growth (negative control, 100% value)

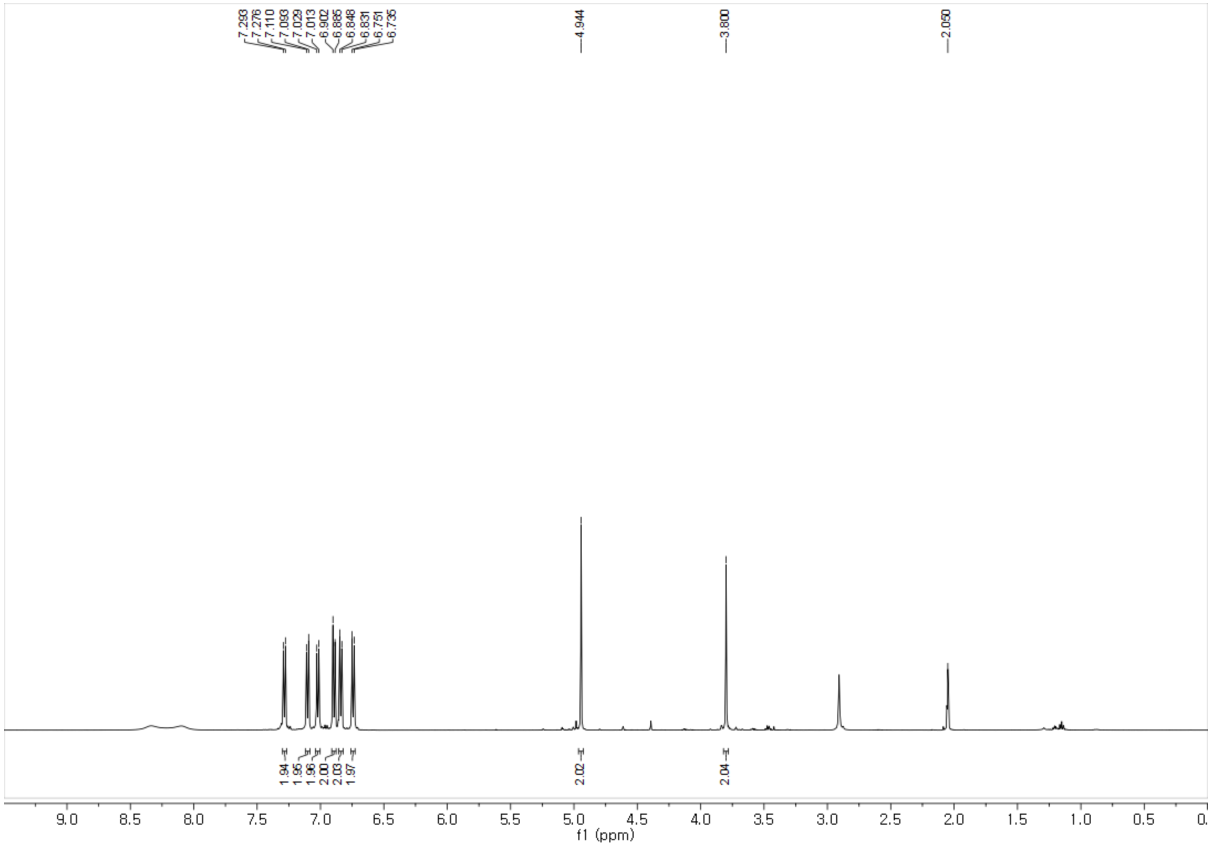

Figure S1. The ^{1}H NMR spectrum of compound **1** (500 MHz, CD_3COCD_3).

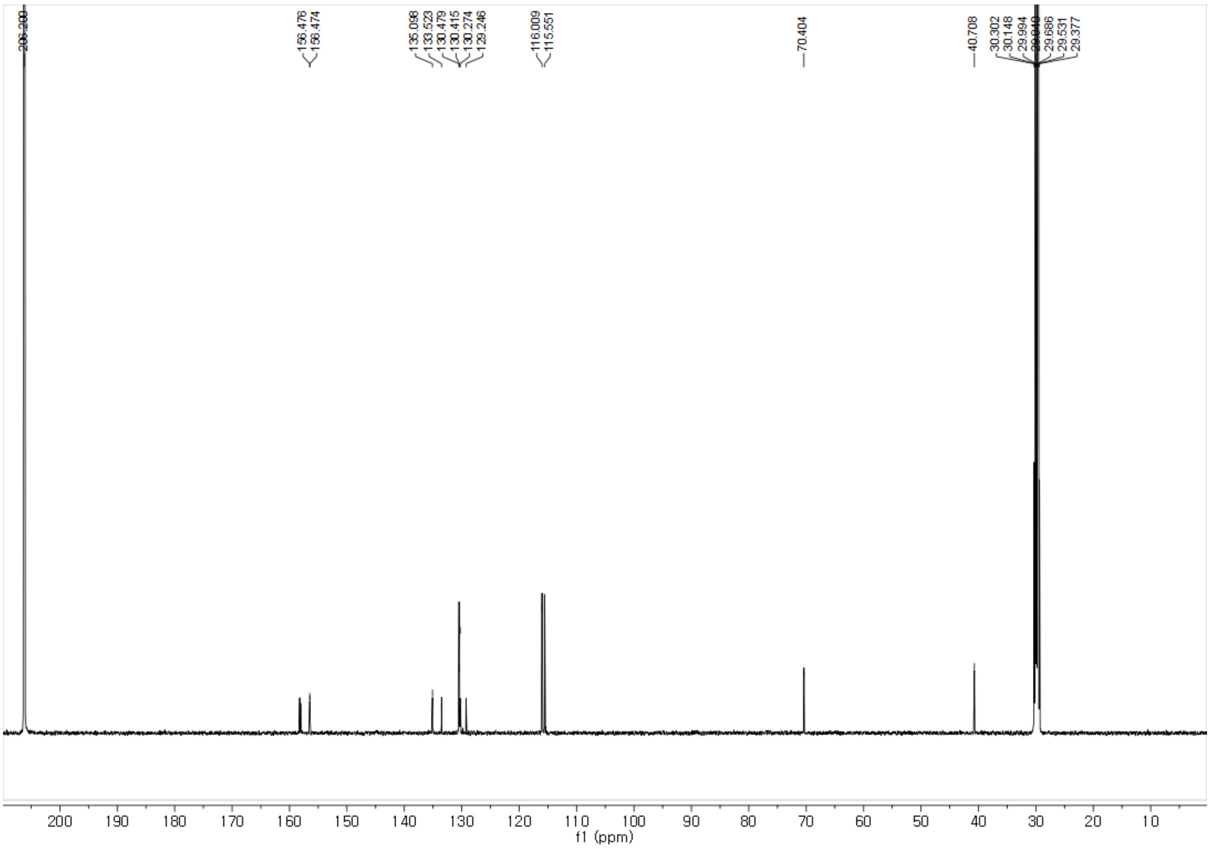

Figure S2. The ^{13}C NMR spectrum of compound 1 (125 MHz, CD_3COCD_3).

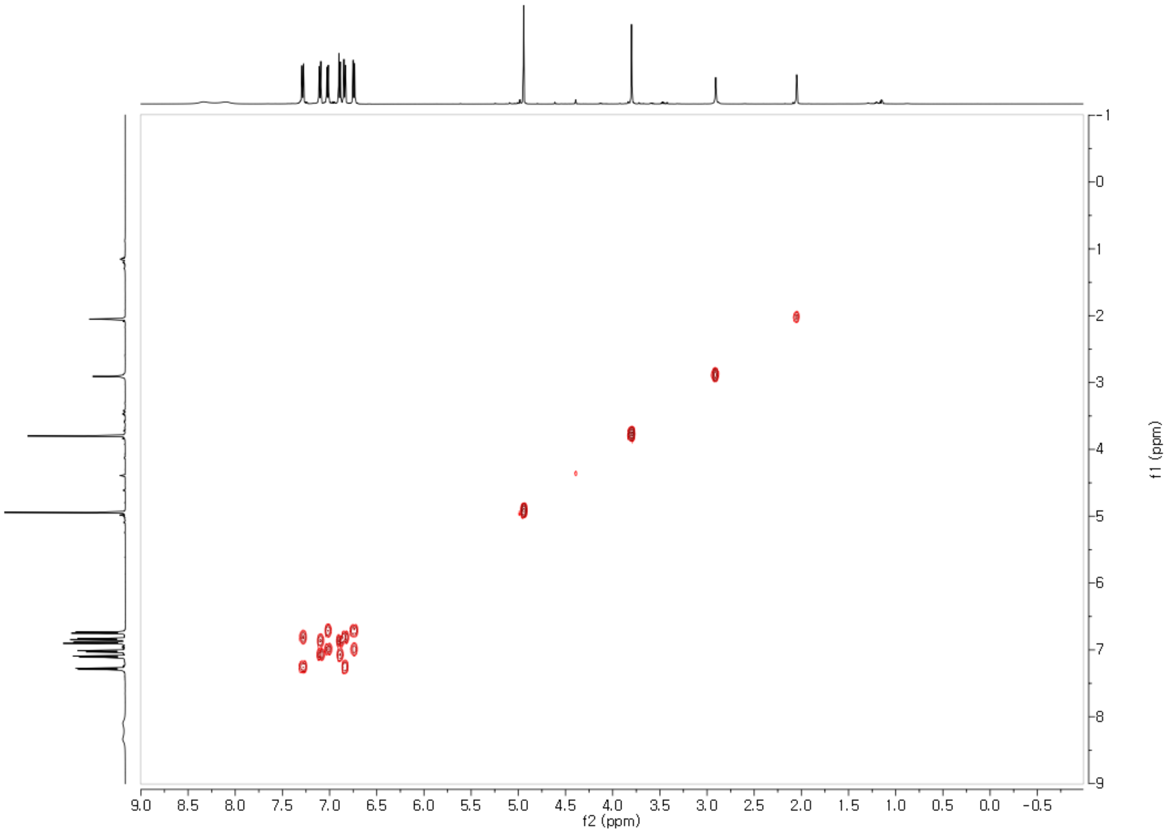

Figure S3. The ^{1}H-^{1}H COSY NMR spectrum of compound **1** (500 MHz, CD_3COCD_3).

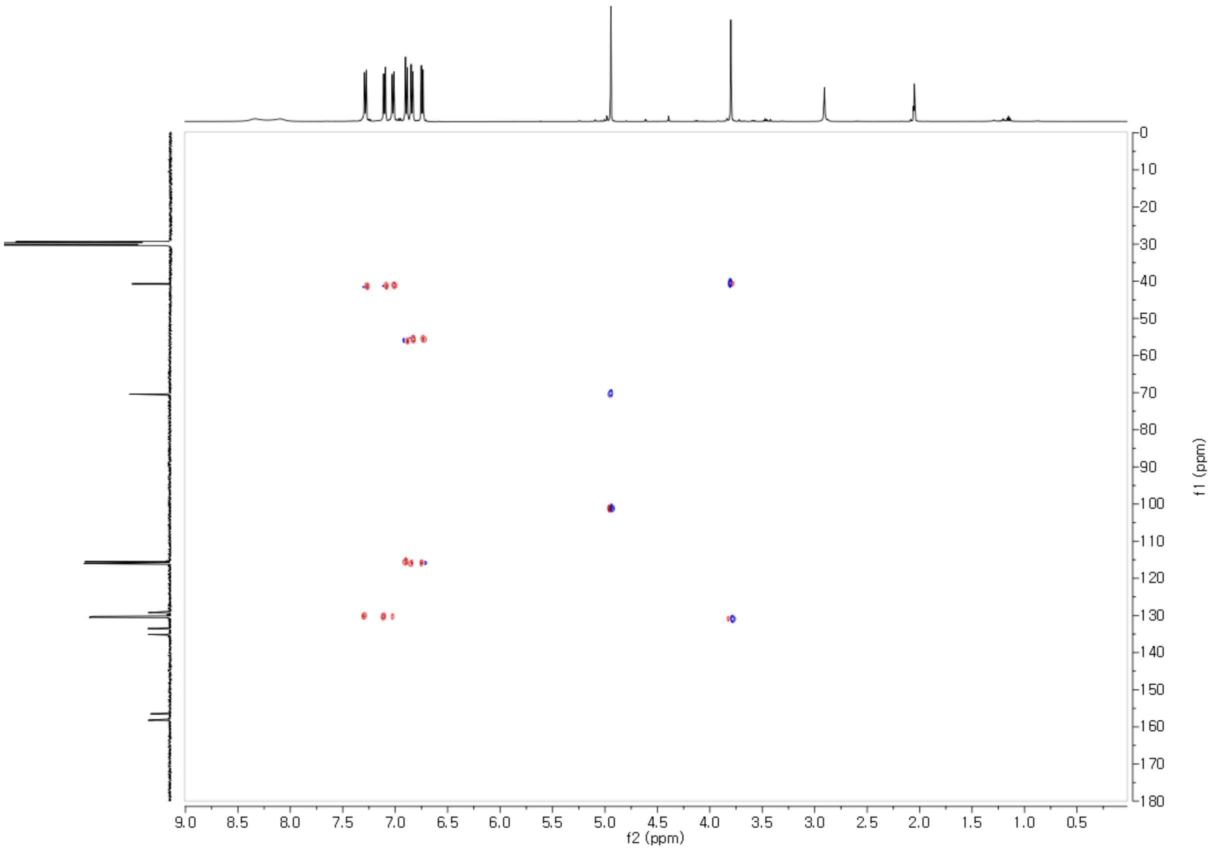

Figure S4. The HSQC NMR spectrum of compound **1** (500 MHz, CD_3COCD_3).

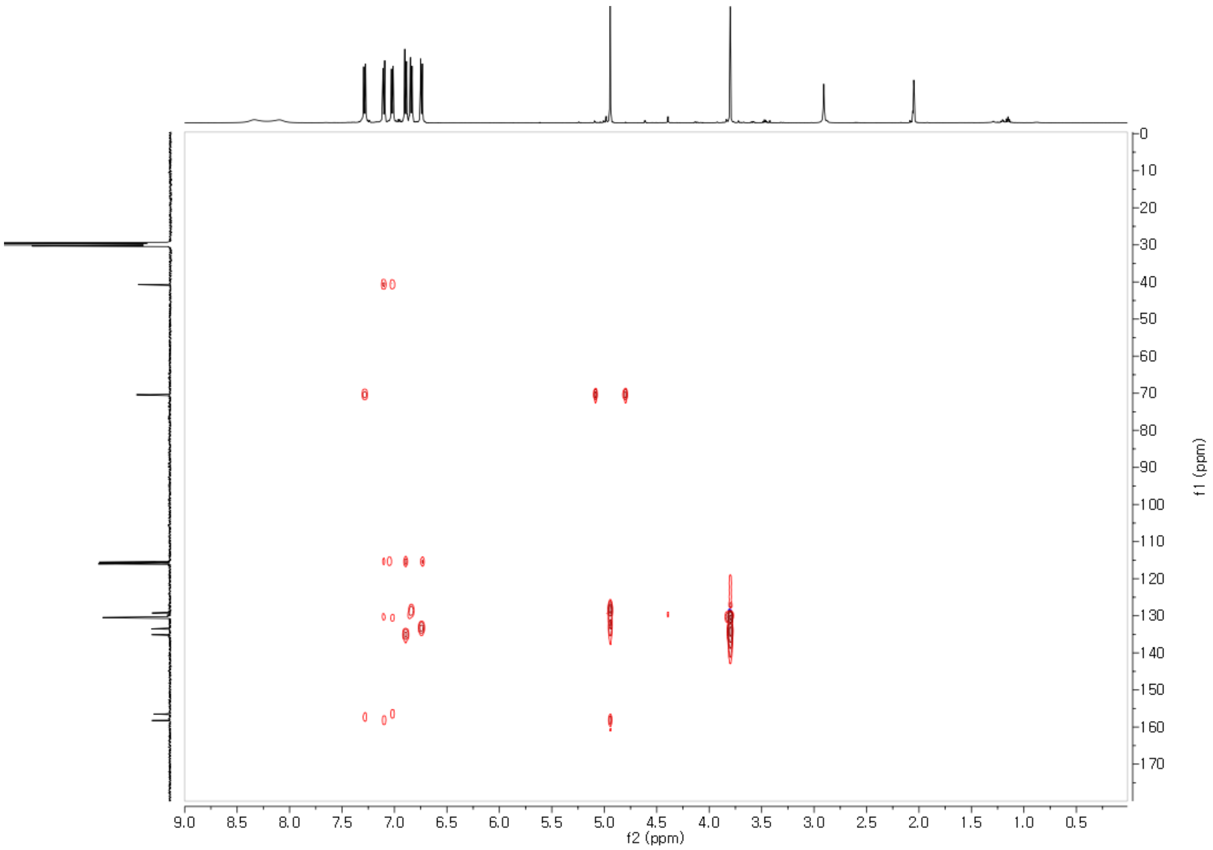

Figure S5. The HMBC NMR spectrum of compound **1** (500 MHz, CD_3COCD_3).

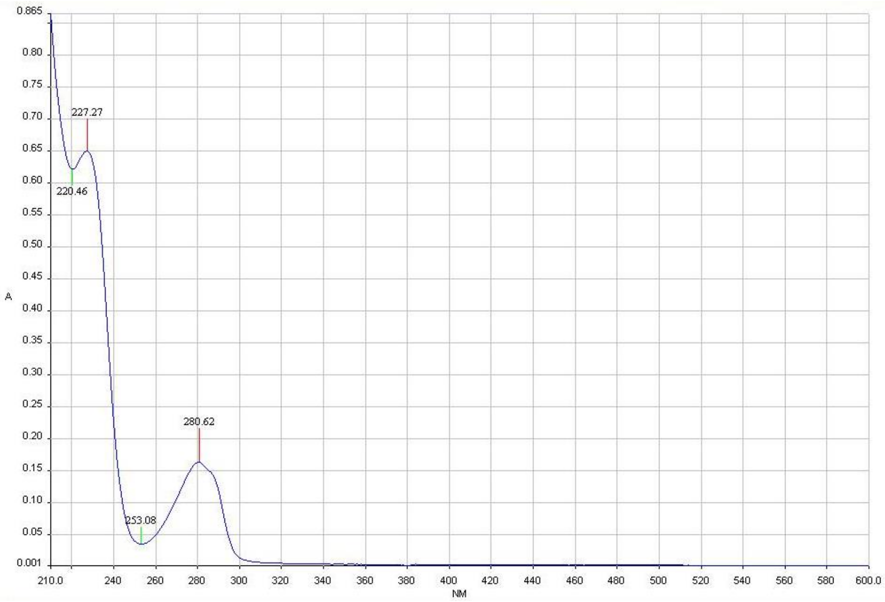

Figure S6. The UV spectrum of compound 1 (CH_3OH, $c=1.6 \times 10^{-5}$ M).

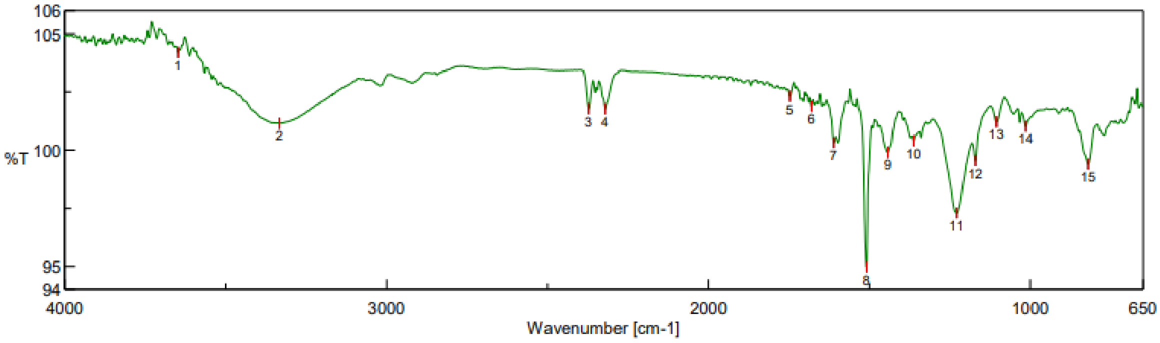

[Result of Peak Picking]

No.	Position	Intensity	No.	Position	Intensity	No.	Position	Intensity
1	3648.66	104.195	2	3334.32	101.142	3	2372.98	101.777
4	2320.91	101.772	5	1747.19	102.315	6	1679.69	101.887
7	1611.23	100.316	8	1509.03	94.9727	9	1442.49	99.8964
10	1362.46	100.389	11	1228.43	97.2937	12	1170.58	99.5382
13	1105.01	101.215	14	1014.37	101.023	15	820.563	99.4001

Figure S7. The IR spectrum of compound **1** [using the attenuated total reflection (ATR) sampling technique].

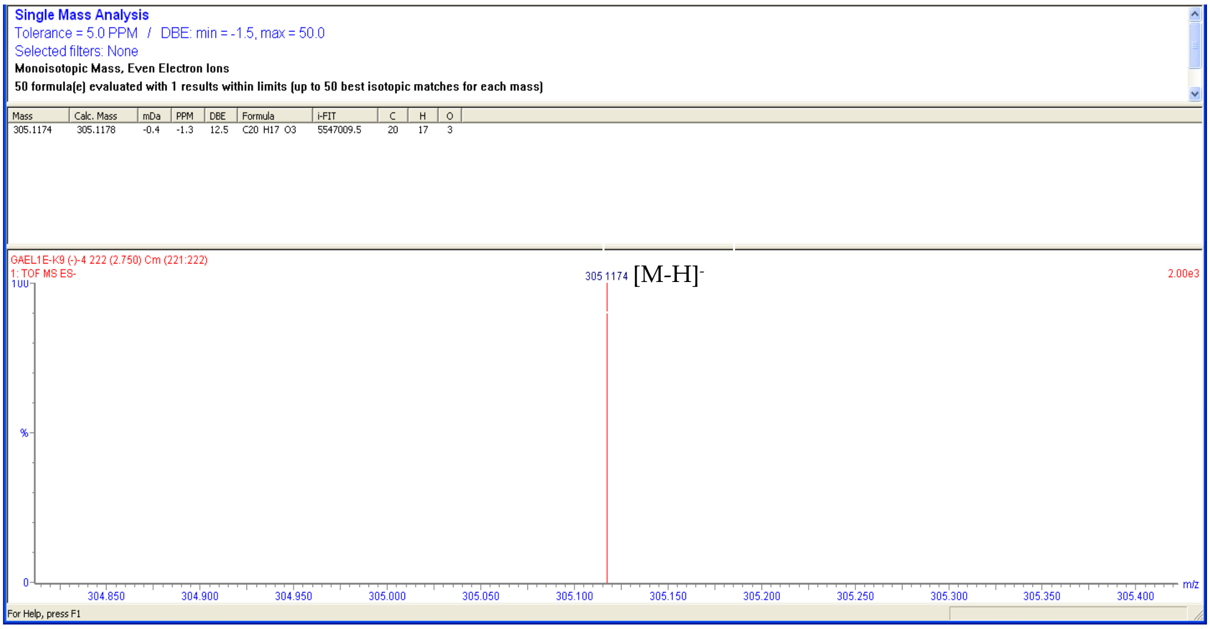

Figure S8. The HRESIMS spectrum of compound **1** (*m*/*z* 305.1174 [M – H]⁻; calcd for $C_{20}H_{17}O_3$, 305.1178).

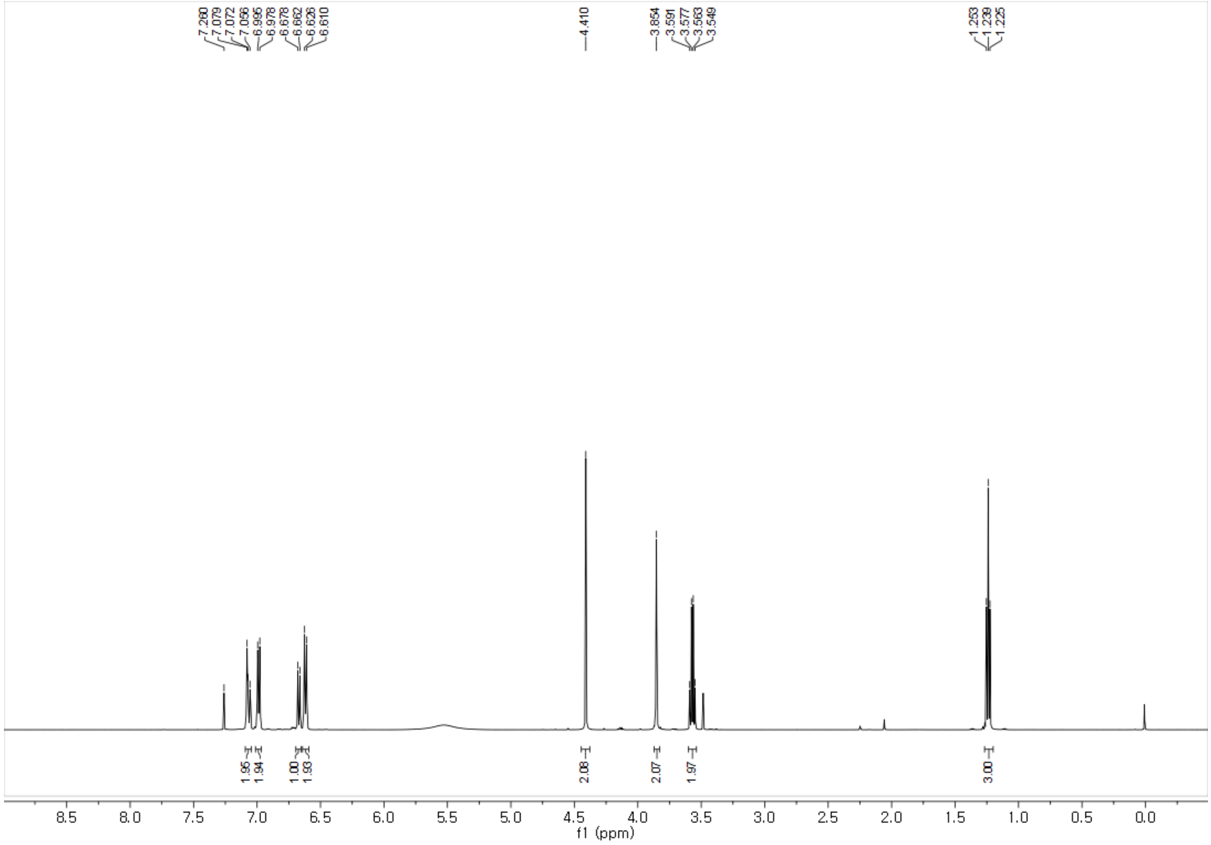

Figure S9. The ^{1}H NMR spectrum of compound **2** (500 MHz, $CDCl_3$).

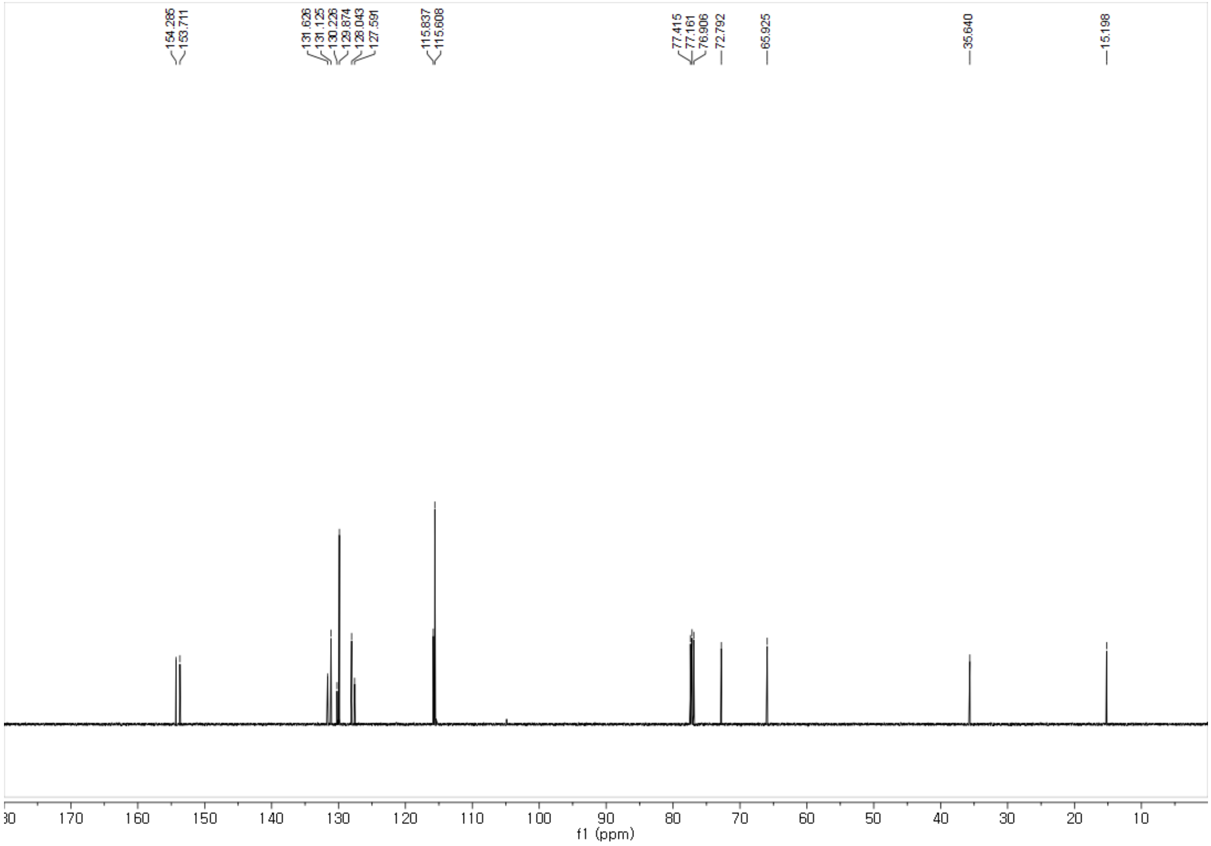

Figure 10. The ^{13}C NMR spectrum of compound **2** (125 MHz, $CDCl_3$).

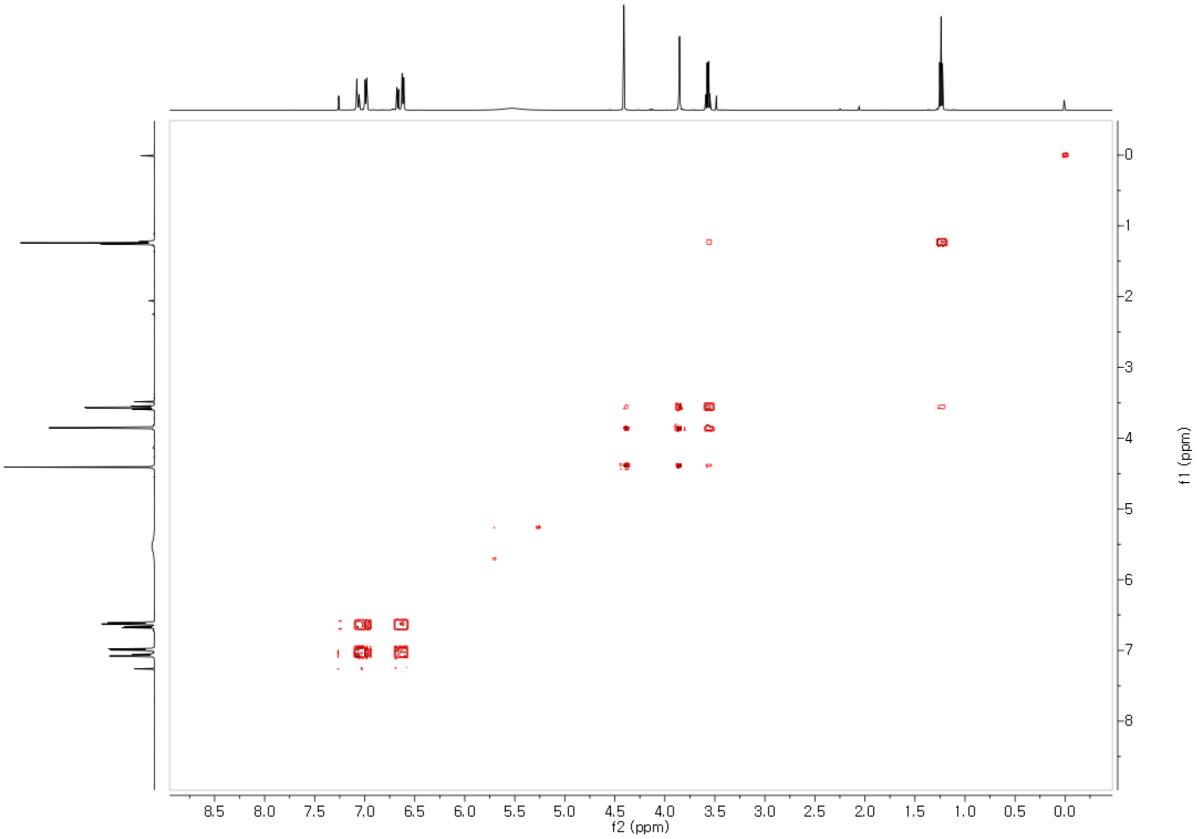

Figure S11. The ^{1}H-^{1}H COSY NMR spectrum of compound **2** (500 MHz, $CDCl_3$).

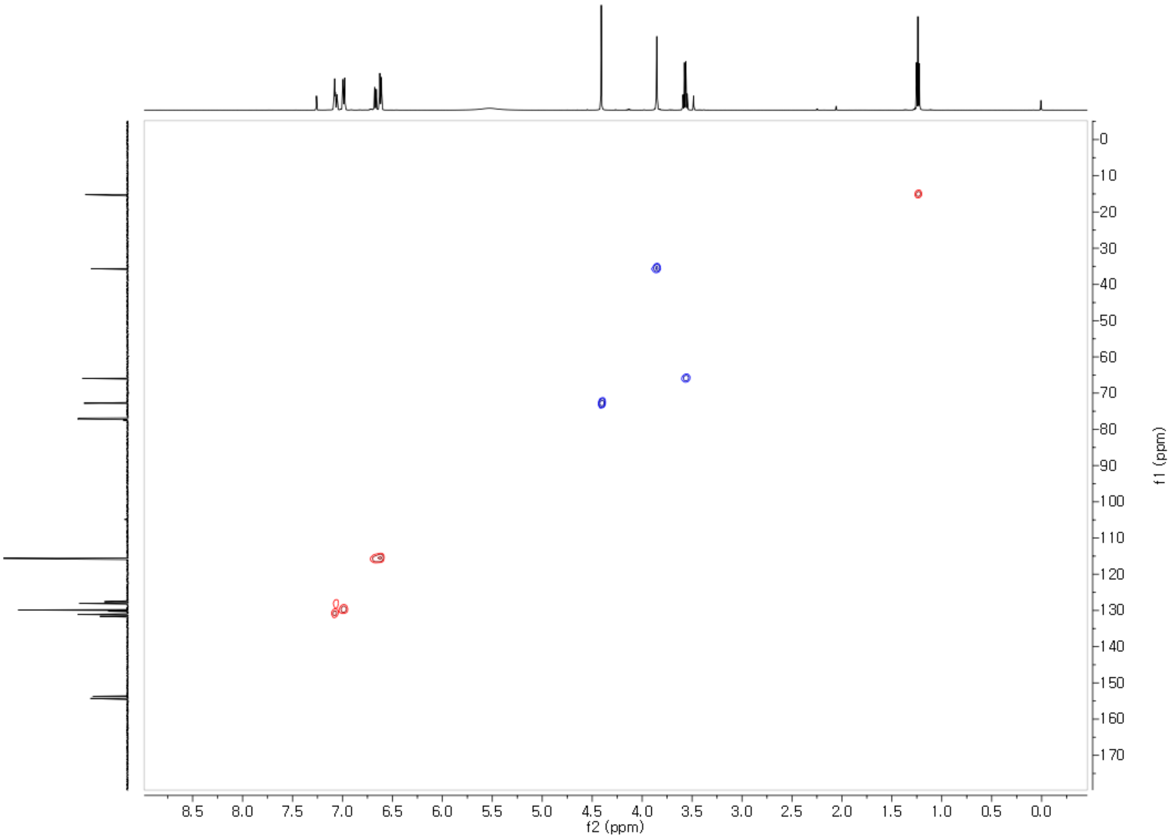

Figure S12. The HSQC NMR spectrum of compound **2** (500 MHz, $CDCl_3$).

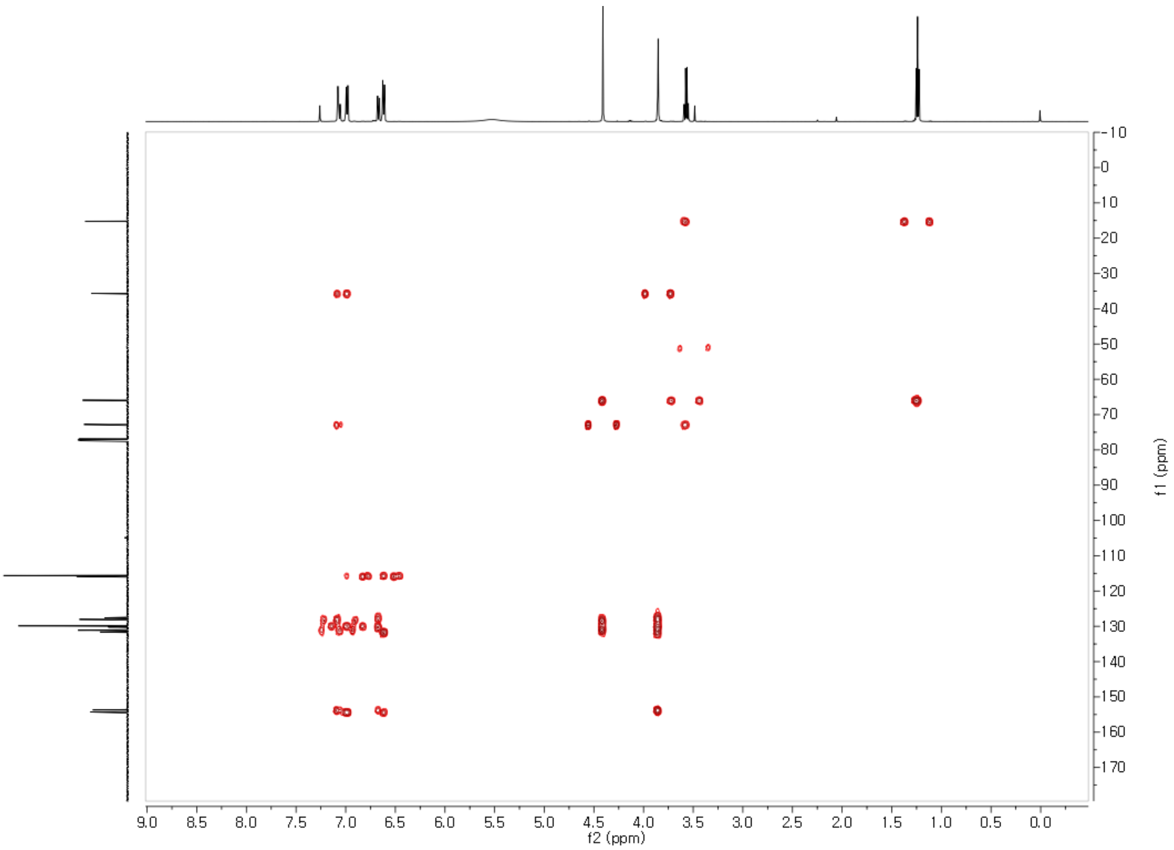

Figure S13. The HMBC NMR spectrum of compound **2** (500 MHz, $CDCl_3$).

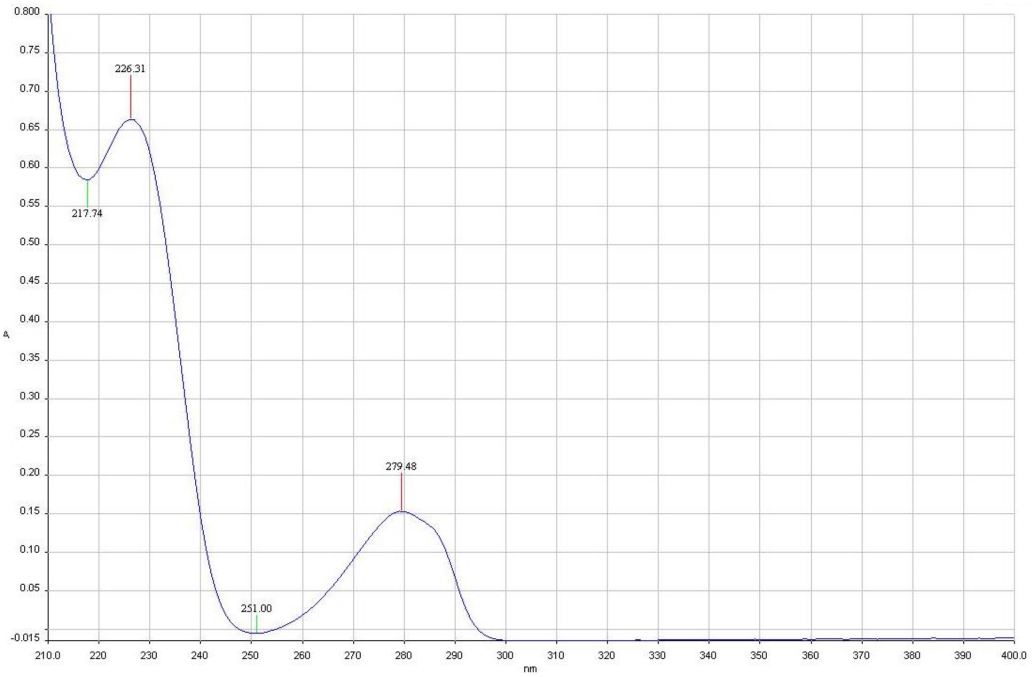

Figure S14. The UV spectrum of compound **2** (CH_3OH, c=1.9 × 10^{-5} M).

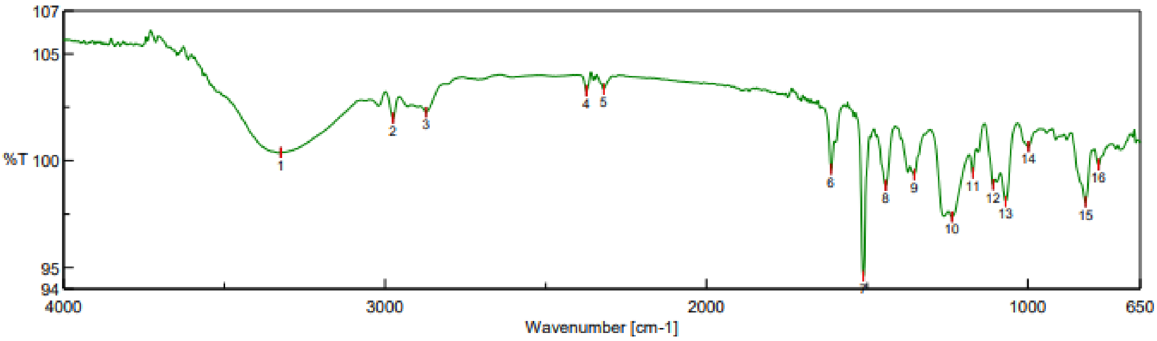

[Result of Peak Picking]

No.	Position	Intensity	No.	Position	Intensity	No.	Position	Intensity
1	3324.68	100.355	2	2974.66	101.982	3	2872.45	102.262
4	2373.94	103.259	5	2319.95	103.336	6	1612.2	99.5903
7	1511.92	94.5818	8	1441.53	98.8363	9	1352.82	99.3367
10	1236.15	97.3676	11	1171.54	99.4209	12	1107.9	98.8775
13	1069.33	98.0915	14	997.982	100.653	15	820.563	98.0665
16	780.065	99.8064						

Figure S15. The IR spectrum of compound **2** [using the attenuated total reflection (ATR) sampling technique].

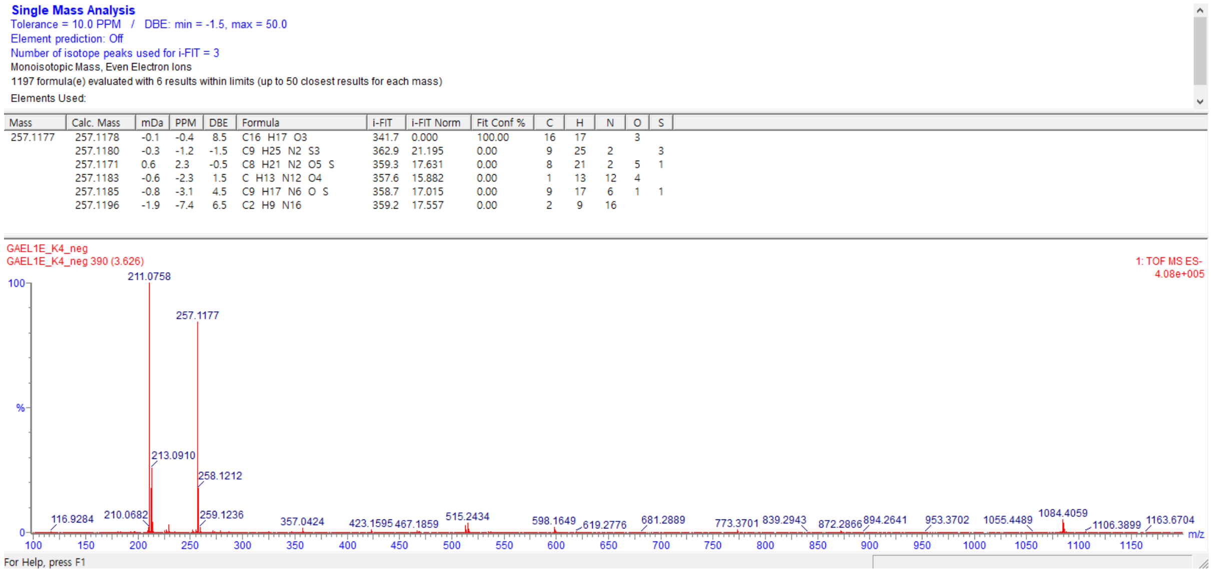

Mass	Calc. Mass	mDa	PPM	DBE	Formula	i-FIT	i-FIT Norm	Fit Conf %	C	H	N	O	S
257.1177	257.1178	-0.1	-0.4	8.5	C16 H17 O3	341.7	0.000	100.00	16	17		3	
	257.1180	-0.3	-1.2	-1.5	C9 H25 N2 S3	362.9	21.195	0.00	9	25	2		3
	257.1171	0.6	2.3	-0.5	C8 H21 N2 O5 S	359.3	17.631	0.00	8	21	2	5	1
	257.1183	-0.6	-2.3	1.5	C H13 N12 O4	357.6	15.882	0.00	1	13	12	4	
	257.1185	-0.8	-3.1	4.5	C9 H17 N6 O S	358.7	17.015	0.00	9	17	6	1	1
	257.1196	-1.9	-7.4	6.5	C2 H9 N16	359.2	17.557	0.00	2	9	16		

Figure S16. The HRESIMS spectrum of compound **2** (*m/z* 257.1177 $[M - H]^-$; calcd for $C_{16}H_{17}O_3$, 257.1178).

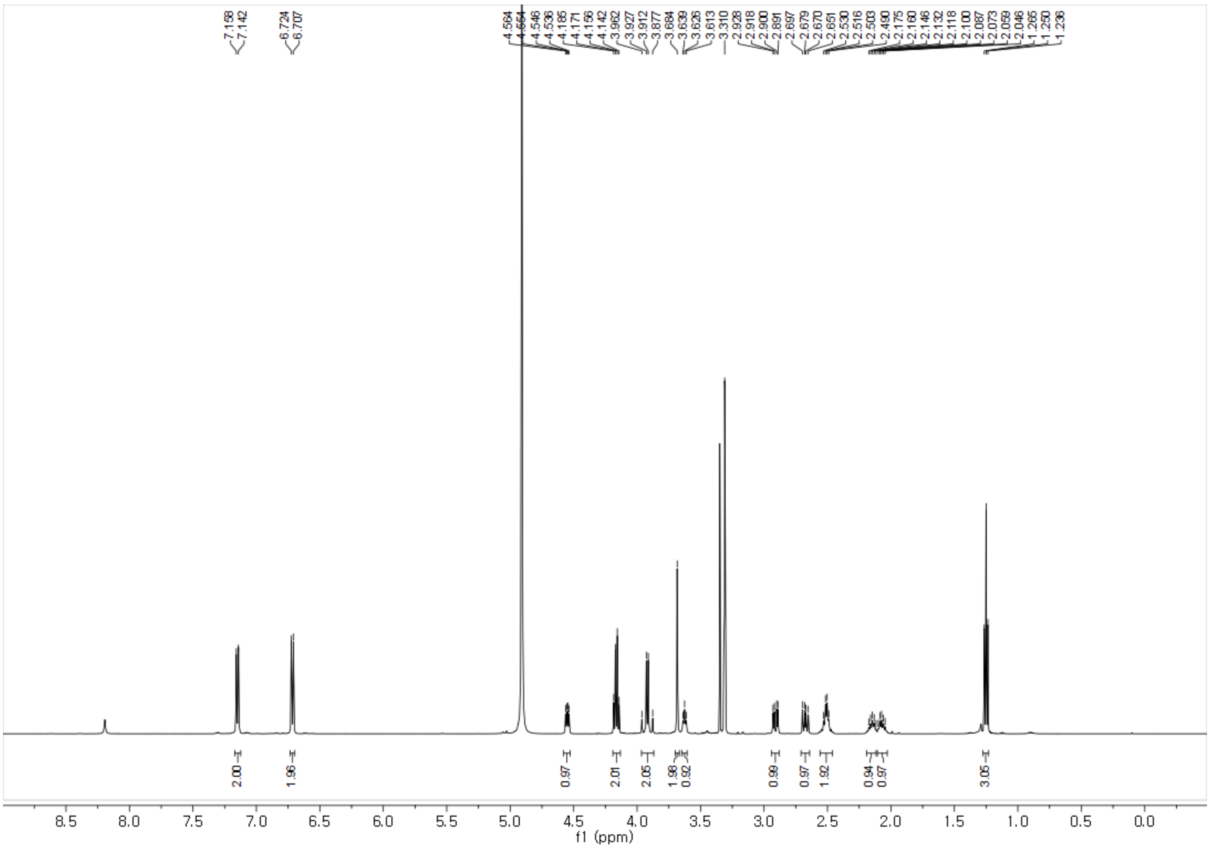

Figure S17. The ^{1}H NMR spectrum of compound **3** (500 MHz, CD_3OD).

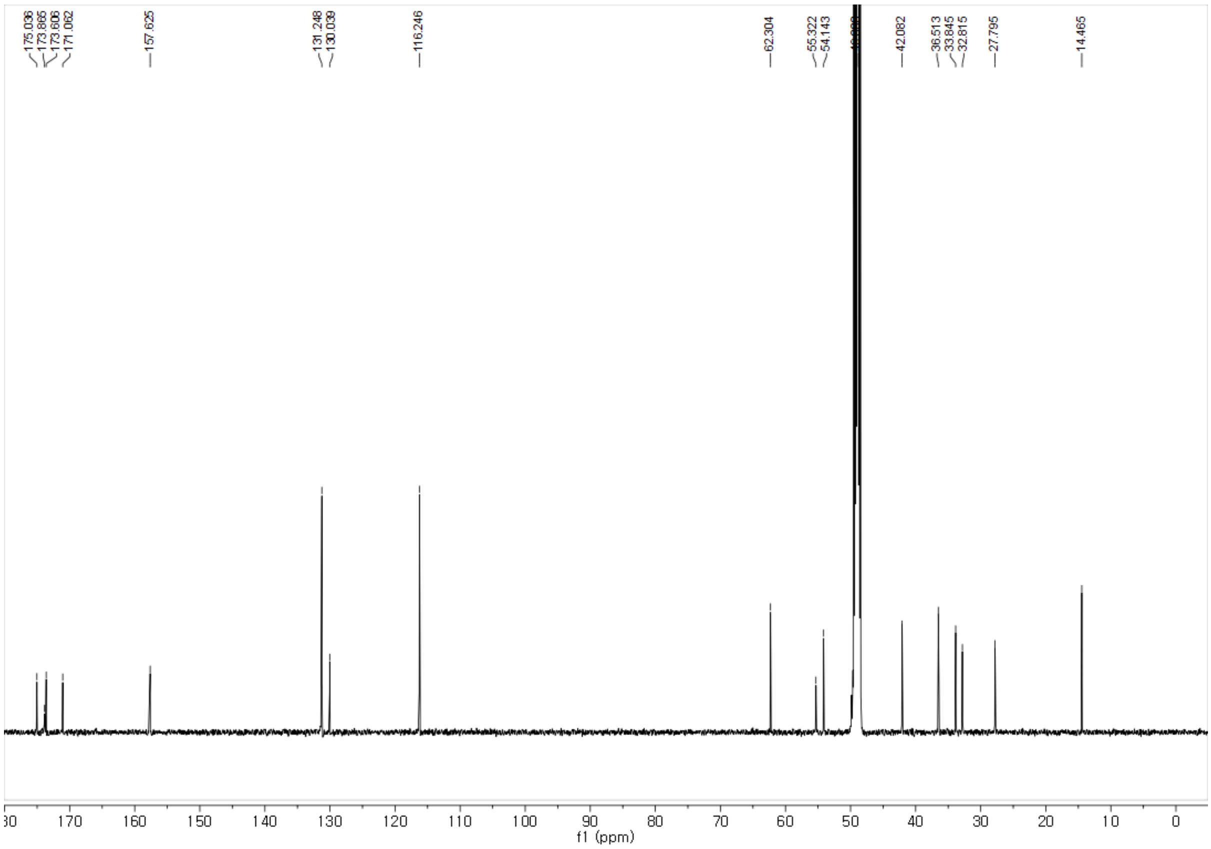

Figure S18. The ^{13}C NMR spectrum of compound **3** (125 MHz, CD_3OD).

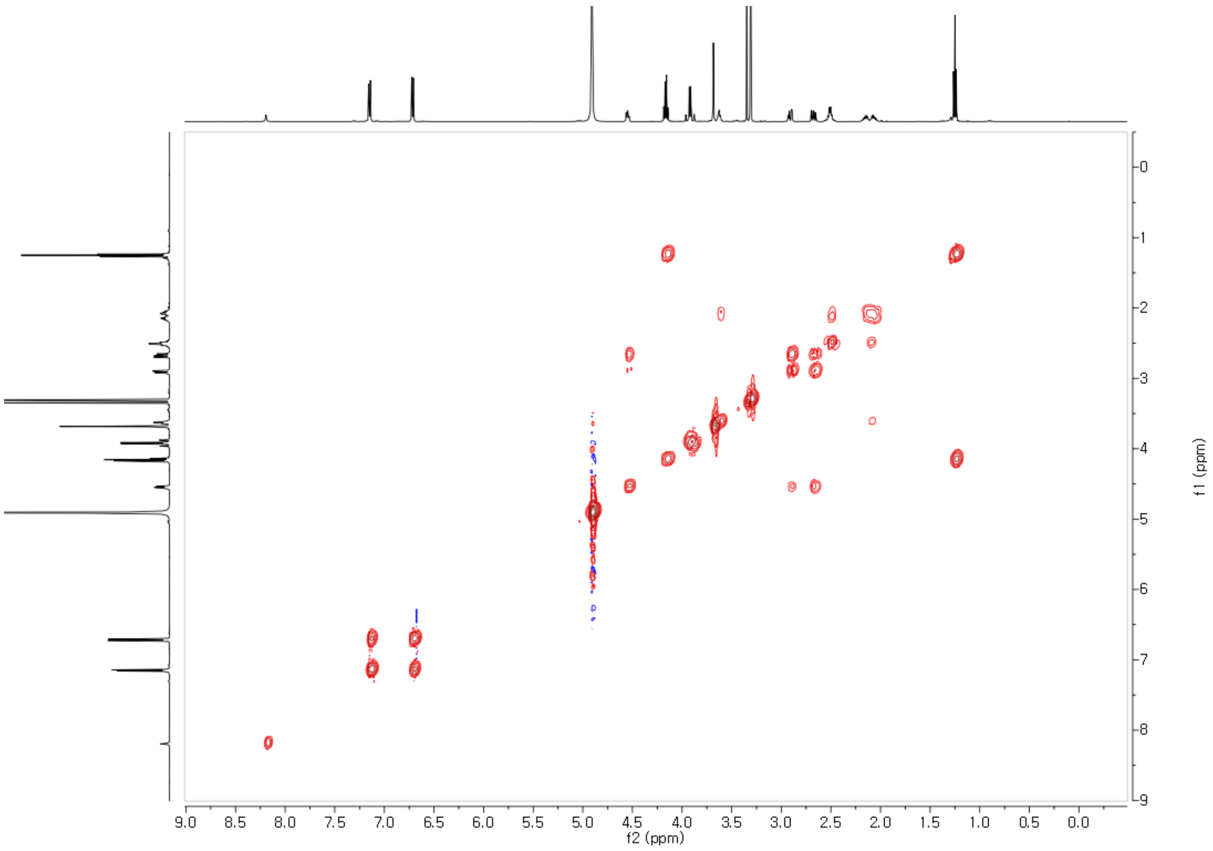

213

214 **Figure S19.** The ^{1}H-^{1}H COSY NMR spectrum of compound **3** (500 MHz, CD_3OD).

215

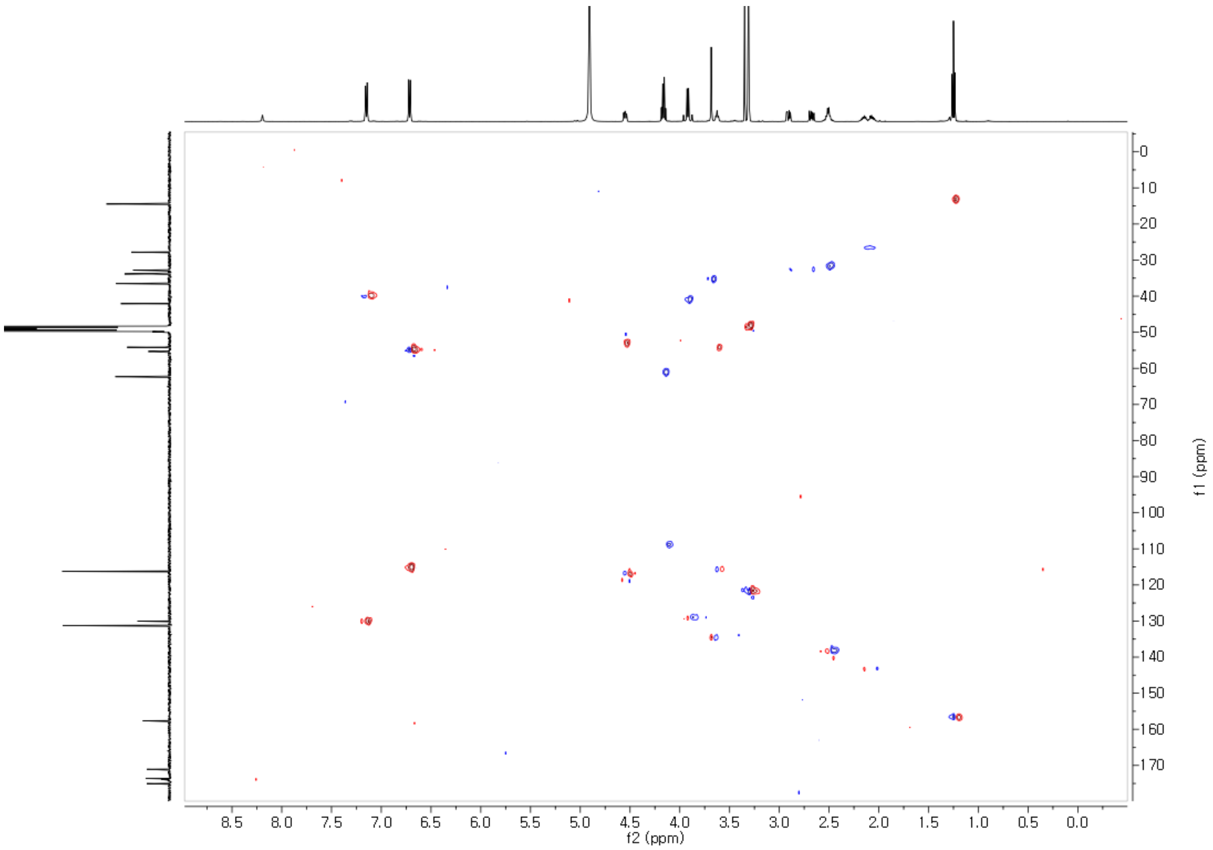

Figure S20. The HSQC NMR spectrum of compound **3** (500 MHz, CD_3OD).

219 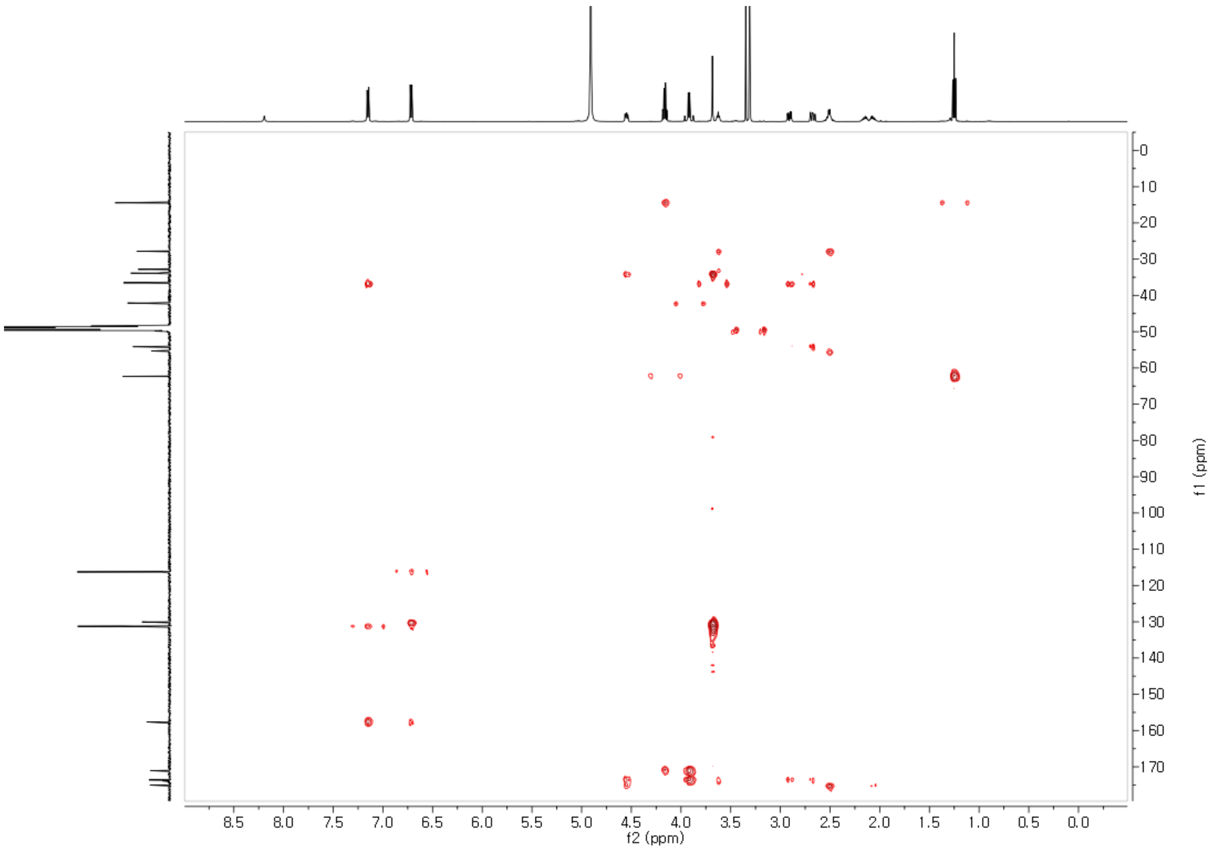

220 **Figure S21.** The HMBC NMR spectrum of compound **3** (500 MHz, CD_3OD).

221

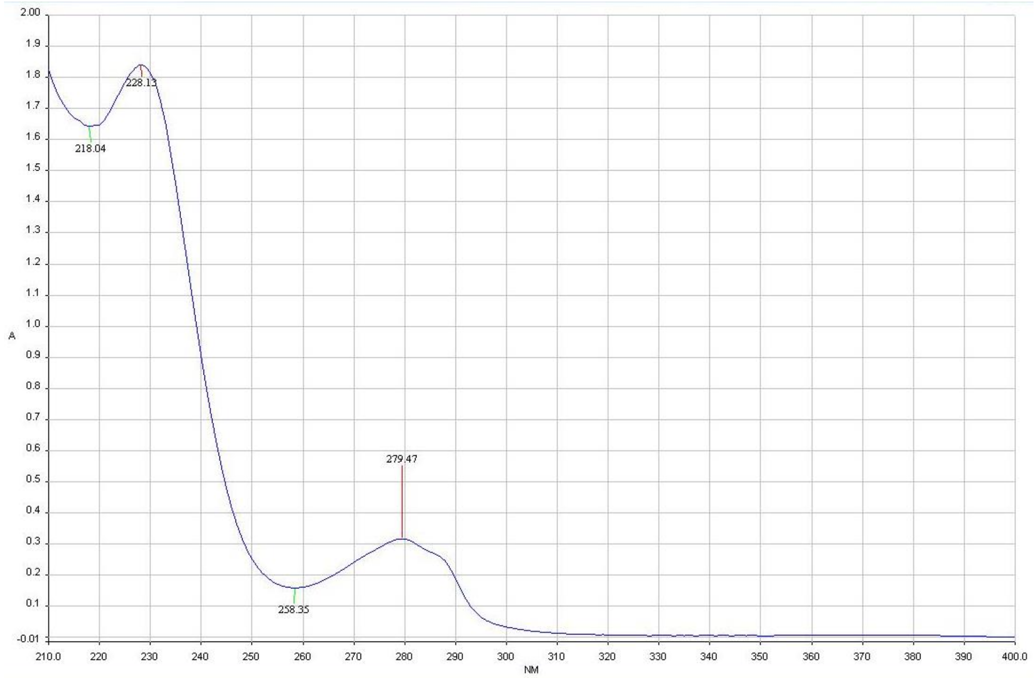

Figure S22. The UV spectrum of compound **3** (CH_3OH, c=1.1 × 10^{-5} M).

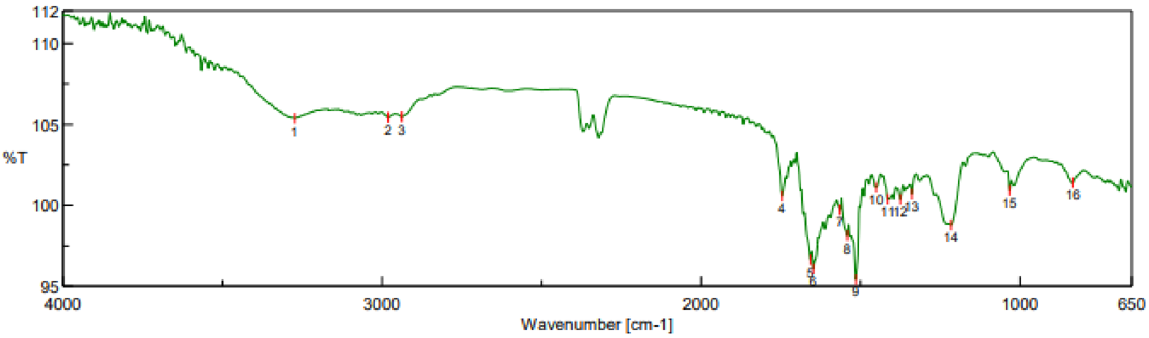

[Result of Peak Picking]

No.	Position	Intensity	No.	Position	Intensity	No.	Position	Intensity
1	3273.57	105.367	2	2981.41	105.429	3	2938.02	105.465
4	1746.23	100.575	5	1657.52	96.6456	6	1647.88	96.0796
7	1564.95	99.7024	8	1541.81	98.1301	9	1514.81	95.4064
10	1451.17	101.103	11	1415.49	100.378	12	1375.96	100.364
13	1339.32	100.697	14	1216.86	98.7768	15	1032.69	100.914
16	835.026	101.395						

Figure S23. The IR spectrum of compound **3** [using the attenuated total reflection (ATR) sampling technique]..

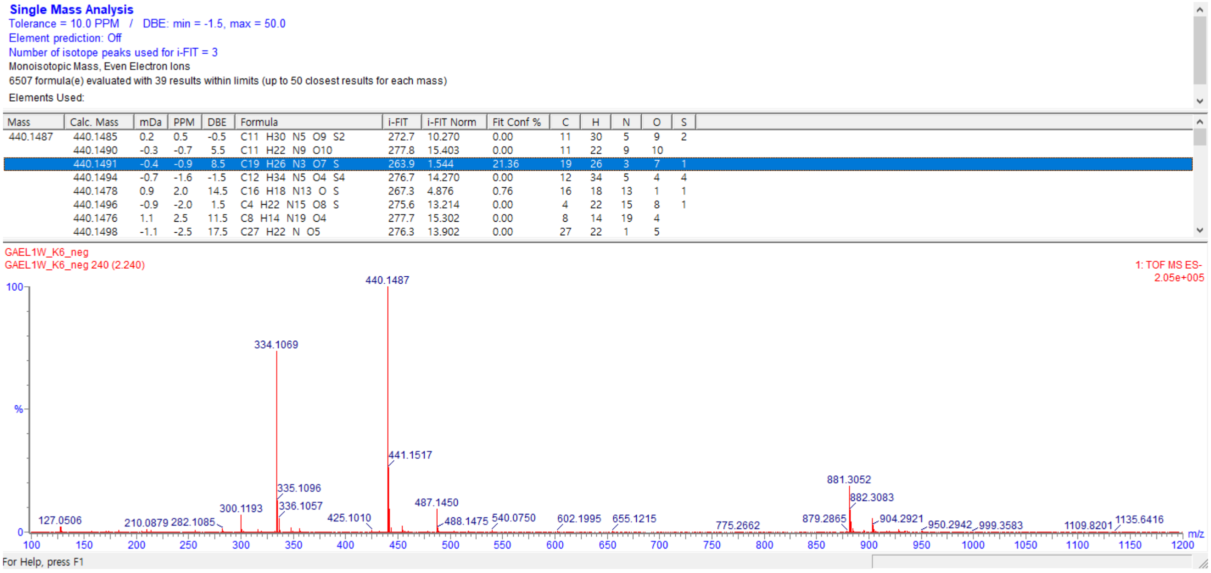

Figure S24. The HRESIMS spectrum of compound **3** (*m/z* 440.1487 [M – H]⁻; calcd for $C_{19}H_{26}N_3O_7S$, 440.1491).
